# Vaccinia Virus Natural Infections in Brazil: The Good, the Bad, and the Ugly

**DOI:** 10.3390/v9110340

**Published:** 2017-11-15

**Authors:** Jaqueline Silva de Oliveira, Poliana de Oliveira Figueiredo, Galileu Barbosa Costa, Felipe Lopes de Assis, Betânia Paiva Drumond, Flávio Guimarães da Fonseca, Maurício Lacerda Nogueira, Erna Geessien Kroon, Giliane de Souza Trindade

**Affiliations:** 1 Laboratório de Vírus, Departamento de Microbiologia, Instituto de Ciências Biológicas, Universidade Federal de Minas Gerais, Belo Horizonte, Minas Gerais 31270-901, Brazil; jaquelinebmedica@hotmail.com (J.S.d.O.); polianaofigueiredo@yahoo.com.br (P.d.O.F.); betaniadrumond@gmail.com (B.P.D.); ernagkroon@gmail.com (E.G.K.); 2 United States Food and Drug Administration, Silver Spring, MD 20993-0002, USA; felipelopesassis@gmail.com; 3 Laboratório de Virologia Básica e Aplicada, Instituto de Ciências Biológicas, Universidade Federal de Minas Gerais, Belo Horizonte, Minas Gerais 31270-901, Brazil; fdafonseca@icb.ufmg.br; 4 Laboratório de Pesquisas em Virologia, Departamento de Doenças Infecciosas e Parasitárias, Faculdade de Medicina de São José do Rio Preto, São José do Rio Preto, São Paulo 15090-000, Brazil; mnogueira@famerp.br

**Keywords:** orthopoxvirus, smallpox vaccine, vaccinia virus, zoonosis, public health, ecology, host range, natural infections

## Abstract

The orthopoxviruses (OPV) comprise several emerging viruses with great importance to human and veterinary medicine, including vaccinia virus (VACV), which causes outbreaks of bovine vaccinia (BV) in South America. Historically, VACV is the most comprehensively studied virus, however, its origin and natural hosts remain unknown. VACV was the primary component of the smallpox vaccine, largely used during the smallpox eradication campaign. After smallpox was declared eradicated, the vaccination that conferred immunity to OPV was discontinued, favoring a new contingent of susceptible individuals to OPV. VACV infections occur naturally after direct contact with infected dairy cattle, in recently vaccinated individuals, or through alternative routes of exposure. In Brazil, VACV outbreaks are frequently reported in rural areas, affecting mainly farm animals and humans. Recent studies have shown the role of wildlife in the VACV transmission chain, exploring the role of wild rodents as reservoirs that facilitate VACV spread throughout rural areas. Furthermore, VACV circulation in urban environments and the significance of this with respect to public health, have also been explored. In this review, we discuss the history, epidemiological, ecological and clinical aspects of natural VACV infections in Brazil, also highlighting alternative routes of VACV transmission, the factors involved in susceptibility to infection, and the natural history of the disease in humans and animals, and the potential for dissemination to urban environments.

## 1. Introduction

The *Poxviridae* family is comprised of large DNA viruses, capable of infecting a variety of organisms. It is divided into two subfamilies: the *Entomopoxvirinae*, which are invertebrate viruses, and the *Chordopoxvirinae*, which are those that infect vertebrates. *Orthopoxvirus* (OPV) is a genus of the *Chordopoxvirinae* subfamily that is significant due to its impact on global public health. Among the 10 species in OPV genus, the variola virus (VARV) is arguably the most significant, due to its role as the etiological agent of smallpox, a devastating human disease [1].

Variola virus (VARV) emerged in the human population concomitantly with the establishment of the first agricultural settlements, probably between 8000 and 10,000 years ago [2]. As the human population began to grow and spread, VARV became endemic in virtually every area of the globe. Smallpox had a profound impact on the human population; responsible for killing approximately 500 million of people in the last century alone [2,3,4,5]. In May 1980, smallpox was declared eradicated due to a herculean effort promoted by the World Health Organization (WHO), which established the global smallpox eradication program. This conquest only became possible due to the worldwide distribution of a live vaccine composed of a related virus from the OPV genus, the vaccinia virus (VACV) [1,3,4,6,7,8]. VACV is remarkably important to the history of vaccination, immunology, and virology. However, despite being an extremely well-studied virus, its origin and natural history remain poorly understood [4].

### 1.1. A Little Bit of History

The concept of vaccination was first explored by Edward Jenner, an English physician, in 1796 [9]. Jenner showed that inoculation of purulent samples from lesions of a pox-infected sick cow (Cowpox) produced a local lesion in the inoculated human, consequently protecting against smallpox. The vaccination process was then created and the virus used, cowpox virus (CPXV), was arm-to-arm transported to different countries and continents [3,4,10]. Stimulated by the fear of vaccinal syphilis transmission from human sources of the vaccine, the arm-to-arm vaccination was then replaced by propagation of the vaccine in calves in the mid-19th century [3]. Calves were first used for vaccine production in Italy, and this process gradually spread throughout Europe [3]. By the end of the 19th century, there several “backyard” factories had been established in Europe where vaccine production was carried out as an unregulated activity. Due to the lack of quality control and regulation unsurprisingly, the “vaccine virus” was, in fact, a miscellaneous mixture of different virus strains of different origins, passage histories, and properties [3,4].

The first description of VACV appeared in historical descriptions of the smallpox vaccination process, and date back to the 1930s, when Allan Downie demonstrated that the material used for the smallpox vaccination (vaccinia virus) at that time had distinct biological properties that were distinct from the cowpox virus (CPXV) [3,11,12,13]. VACV had been introduced randomly over the course of vaccine manufacture in the 18th or 19th centuries [13]. At that time, arm-to-arm vaccination had already been replaced by propagation in calves.

The development of a heat stable vaccine was only achieved in the 20th century. By 1950, the vaccine was produced by vaccine manufacturers who infected the flanks of calves with vaccinia [4]. In 1953, the global eradication program was proposed by the WHO, however, no country expressed interest in eradicating smallpox worldwide until 1958. It was only in 1966, during the 19th WHO World Health Assembly, that the global eradication program took off due to increased investment in this effort [3,4,14].

By February 1967, there were 77 vaccine manufacturers distributed throughout 52 countries. At that time the vaccine was produced by methods which included multiple hosts for virus growth including calf, sheep, water buffalo, chick embryo and tissue culture (bovine embryo fibroblasts culture). The vaccines produced on the skin of animals were by far the most extensively used by manufacturers throughout the world, including developed and developing countries. During the Intensified Smallpox Eradication Programme, launched in 1967, several VACV strains were used worldwide, which included the Lister, New York City Body of Health and Paris strains, and it is probable that some of these strains shared a common ancestry. These vaccine strains were chosen based on the low virulence profile and their distribution was varied between the different continents [3].

**Figure 1:**
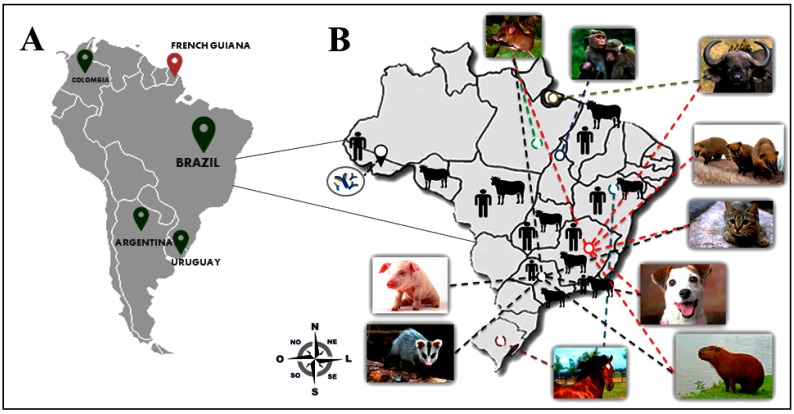
Detection and distribution of vaccinia virus (VACV) in South America. (**A**) A map of South America is shown on the left and the green pins indicate countries where VACV has been detected in recent years. The red pin indicates the absence of VACV detection in French Guiana; (**B**) A map of Brazil highlighting the distribution of VACV in different regions and the detection of VACV in a broad range of hosts. The antibodies (blue) indicate serological evidence of VACV circulation in humans in rural areas of Acre state. Dashed lines in different colors represent VACV circulation in different Brazilians states, i.e. red dashed lines represent VACV circulation in Minas Gerais State, black dashed lines represent circulation in São Paulo State, and green dashed lines represent circulation in Pará State.

Among the orthopoxviruses, VACV is the most comprehensively studied. During the time of mass vaccination, it was assumed that VACV could never establish itself in nature due to the fact that it had presumably become laboratory attenuated and because its origin and natural hosts remained obscure [1,13,15,16,17,18]. However, several VACV strains have been described from different locations throughout the world. In India, a VACV sub-lineage, named buffalopox virus (BPXV), is a re-emerging zoonotic viral infection affecting mainly buffaloes, but also bovines, and humans that have come into direct contact with those infected animals [19,20,21]. The first recorded incidence of BPXV infection in buffaloes was in 1934 [22,23,24,25,26], and since then several outbreaks have been described in India but also in other countries such as Pakistan, Egypt, Nepal, and Bangladesh [19,20,27,28]. In addition, another VACV strain, named rabbitpox virus (RPXV), has been associated with infections in domestic rabbits in the Netherlands and the United States [29,30]. The disease ecology and virus transmission chain for these strains are still poorly understood. Similarly to the Indian subcontinent, the circulation of VACV has been described in some South American countries including: Argentina [31]; Uruguay [32]; Colombia [33], and; especially in Brazil [34] (Figure 1). In this article, we present a comprehensive review of the literature combined with some recent data obtained by our research group focused on the emergence of VACV in Brazil due to the preponderance of cases reported, and also because of the impact that its natural circulation is causing in this country.

The circulation of zoonotic VACV was first reported in Southeast Brazil in 1999 [35,36]. The infection was associated with several exanthematous outbreaks that have been described in Brazilian rural areas affecting mainly milking cattle and their handlers [37,38,39,40,41,42,43,44,45,46]. In Brazil, the disease caused by VACV is popularly known as “bovine vaccinia” (BV), probably due to the fact that most cases have been described in dairy cattle. In this review, we will focus on the emergence of VACV in South America, mainly in Brazil, addressing the clinical, evolutionary and eco-epidemiological aspects.

### 1.2. The Disease Named Bovine Vaccinia

**Figure 2:**
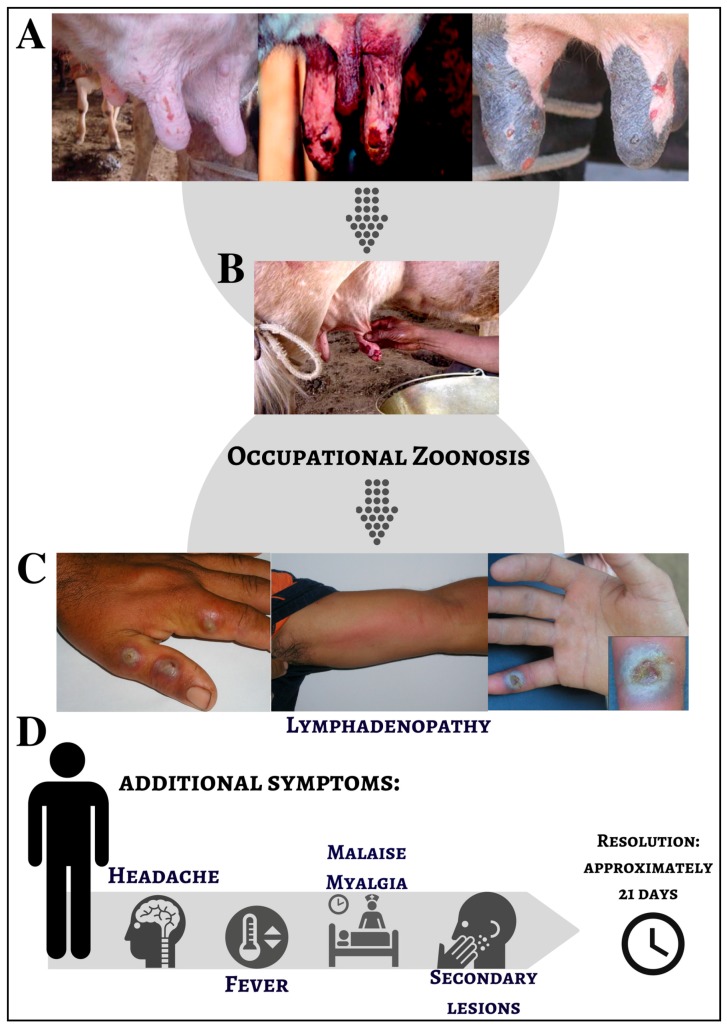
Clinical presentation of bovine vaccinia infection in dairy cattle and humans. (**A**) Nodular and ulcerative lesions on the udder and teats of dairy cows; (**B**) The classical transmission of VACV involves direct contact between dairy workers and infected cows; (**C**) Nodular and ulcerative lesions on hands and forearms of rural workers; (**D**) Additional systemic symptoms present during VACV infection in humans (Source: [43,53,54,55]).

Bovine vaccinia (BV) is the name used to describe a vesiculopustular exanthematous disease in milking bovine herds and dairy workers who are in direct contact with these animals. BV is characterized by exanthematic lesions on the teats and udders of affected cows [38,47,48,49,50]. In naturally infected cows, BV lesions appear as red papules mainly located on the skin of the teats and udder. Papules progress to vesicles and vesicles become umbilicated pustules surrounded by inflammatory tissue. These pustules progress to ulcers until complete wound healing occurs (Figure 2). Calves that feed on infected cows often present with lesions on the lips, muzzle, and mouth [38,51]. A recent study has shown that in experimentally infected dairy cows, vesicles and papules appear around 3–4 days post infection (d.p.i.), and ulcers appear around the 5th d.p.i. and can last up to the 15th d.p.i. Scab formation begins on the 6th d.p.i., and healing commences on the 18th d.p.i. Infected dairy cows can develop well-defined ulcers in the oral mucosa and the mammary lymph nodes can appear enlarged by the 18th d.p.i. [47,48,49,52].

BV is considered an occupational zoonosis, with dairy workers and farmers being the main risk group. Studies reporting BV outbreaks have shown that affected individuals generally have direct contact with dairy cattle, often due to the activity of milking. In fact, the processes involved in milking process and basic cattle management represent the main route of VACV transmission [34]. Individuals who have direct contact with infected dairy cows will develop cutaneous localized punctuate lesions on the skin accompanied by itching and followed by local edema and vesicular lesions [38,40,42,43,56,57,58]. Systemic symptoms such as fever, headache, malaise, myalgia, inguinal lymphadenopathy and the development of secondary lesions are also observed (Figure 2) [34,40,42,43,59].

During the intensified vaccination campaign for smallpox eradication in the 1970s, several outbreaks linked to domestic animals derived from vaccinated individuals were reported not only in Brazil but also in other South America countries [3]. Only at the end of the 90s, almost 20 years after the discontinuation of smallpox vaccination in Brazil, BV outbreaks started to emerge (Figure 2) [15,34,60,61].

## 2. The Good: Uncovering the Eco-Epidemiological and Evolutionary Aspects of Natural Infections with Vaccinia Virus

While the emergence of VACV in Brazil has had profound negative impacts on the dairy industry and public health in general, the natural circulation of this virus in the country offers a good opportunity to expand our understanding of its eco-epidemiological and evolutionary aspects.

The first Brazilian VACV isolate dates back to the 1960s and was isolated from wild and sentinel rodents through the efforts of the Rockefeller Institute for Research on Arboviruses located in Brazil [62,63,64] (Figure 1). However, BV outbreaks and VACV isolates from humans and dairy cattle appear in the literature from the late 1990s, from studies based in rural areas of the Southeast region of Brazil (Rio de Janeiro, São Paulo, and Minas Gerais states). While VACV circulation has been reported throughout the country, this southeast region of Brazil remains the epicenter of BV outbreaks, with Minas Gerais state being the most affected region. This is probably due to the fact that Minas Gerais state is the largest producer of milk in the country and possesses some of the largest dairy cattle herds in Brazil [34,65,66,67]. Despite its increasing significance, BV notification is not mandatory to Brazilian health authorities and reports of outbreaks and case studies are still restricted to few research groups in Brazil. While many aspects of natural VACV infection have been described in the last two decades, much is still unknown about its circulation in nature, alternative routes of infection and its natural hosts/reservoirs.

Concomitant with its wide geographical spread, VACV has been detected in a broad range of host species including farming/production animals (bovines, equids, swine, and buffaloes) and companion animals (dogs and cats) [17,34,61,68,69,70,71]. VACV has also been reported in wildlife (Supplementary Table S1) (capybaras, primates, and marsupials) [69,72,73,74] and in synanthropic (*Mus musculus* and *Rattus* sp.) and wild rodents. Despite VACV detection in several mammalian species, viral circulation in sylvatic cycles and the identification of potential reservoirs still represent an aspect of its natural history that requires further exploration [15,18,74,75,76].

In an attempt to elucidate the origin of BV outbreaks and a possible VACV reservoir, some investigators have demonstrated through serological or molecular evidence, OPV and VACV circulation in animals that naturally transit between rural and sylvatic environments. The detection of anti-OPV antibodies and VACV DNA in primates (*Cebus apella* and *Alouatta caraya*) from the Amazon region [72] and procyonids in São Paulo state are good examples [77]. Molecular and serological evidence of VACV circulation has also been observed in several species of marsupials (*Didelphis* sp., and *Caluromys philander*) and wild rodents (*Calomys* sp., *Akodon* sp., *Necromys lasiurus*, *Trinomys setosus*, *Cerradomys subflavus*, *Oligoryzomys* sp., and *Nectomys squamipes*) collected in regions with and without previous BV history [18]. It is important to emphasize that many of the captured rodents are generalists by habit, and therefore, easily adapt to anthropic environments [18]. Indeed, the importance of wild rodents in the VACV transmission chain in rural areas has been suggested, also in conjunction with interactions between wild rodents and marsupials, which could maintain natural VACV circulation and trafficking between forests and peridomestic environments [18]. Furthermore, data presented by Miranda and colleagues reinforce the findings proposed by Abrahão et al., who proposed an ecological model to explain how the rodents could act as a link for VACV spread between wild and anthropic environments [56].

Since the isolation of the first presumably zoonotic VACV isolates in Brazil, two controversial hypotheses have arisen relating to BV outbreaks and the origin of VACV [15,17,75,78]. The first of these proposes that Brazilian vaccinia viruses (Br-VACV) originate from a vaccine strain, which could have escaped to the field. The second hypothesis proposes that these isolates had been circulating among wild animals before the introduction of VACV, though have recently emerged as zoonotic agents. Several studies have confirmed a remarkable two-type population structure following analysis of several VACV isolates, distinguishable by their genetic and biological features, such as molecular signatures (single nucleotide polymorphism (SNP) and insertion or deletion of bases (indels) in hallmark genes), and their virulence in vivo and in vitro [17,68,79,80]. Recently, Medaglia and colleagues [17] proposed a new evolutionary relationship between the Br-VACV isolates and VACV vaccine strains based on molecular analysis of Serro 2 virus (S2V) and Cantagalo virus (CTGV), two Br-VACV isolated during BV outbreaks [35,43]. In that study, the S2V and CTGV were genetically related to VACV Instituto Oswaldo Cruz (VACV-IOC) strain, and not horsepox virus, which is believed to be related to an ancestor of the VACV lineages [13,17]. However, the absence of whole genome sequences from Br-VACV isolates represents a significant gap that would help to elucidate the origin and evolutionary history of the viruses circulating in South America. On the other hand, the characterization of VACV isolated in Colombia during the outbreak in 2014 could provide clues to better understand the evolutionary history of VACV, and also its natural transmission cycle. Usme-Ciro et al. demonstrated that the VACV identified in Colombia were related to Br-VACV group 1 isolates, although phylogenetic analysis suggests that the strains from Brazil and Colombia diverged long ago or independently arose [33].

**Figure 3:**
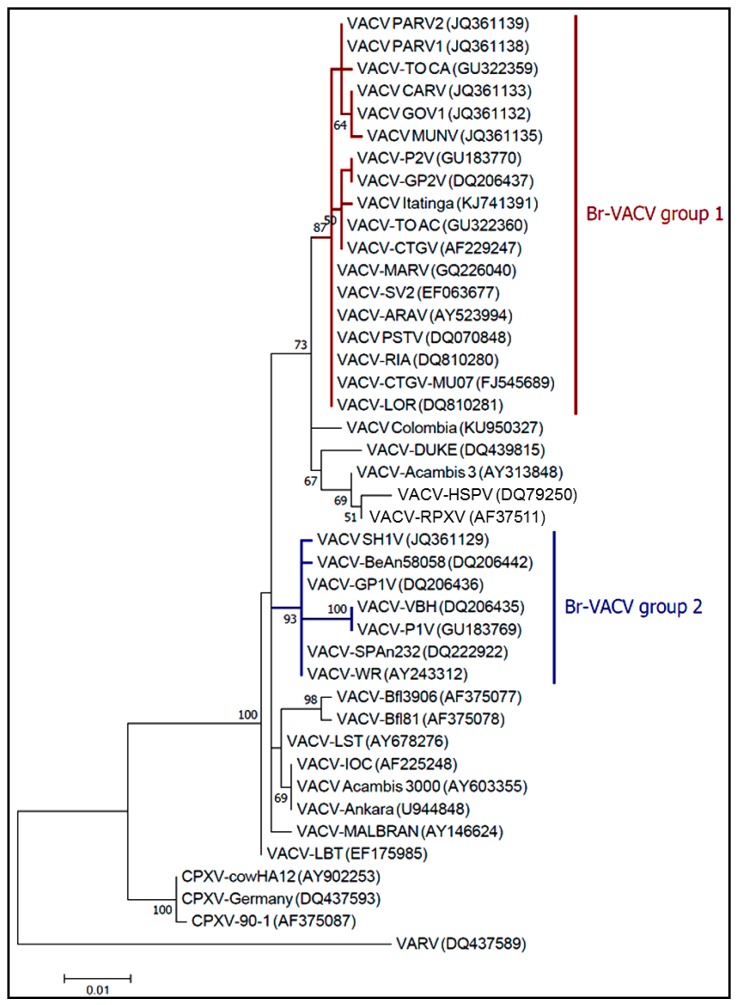
Phylogenetic analysis based on the A56R gene of VACV vaccine and wild isolates, with cowpox virus (CPXV) sequences included as an outgroup. These sequences are available in the NCBI nucleotide database under the GenBank Accession Numbers provided in brackets on the tree. The sequences were aligned by using ClustalW algorithm and the evolutionary history was inferred by using the Maximum likelihood (ML) method, using Mega 7.0 (GE Healthcare, Buckinghamshire, UK) software and the Jukes-cantor model was selected for ML inference by the program JmodelTest 2.1.6 (Free Software Foundation, Inc., Boston, MA) The evolutionary distances were computed using the Maximum Composite Likelihood method with 1000 Bootstrap replicates. The analysis involved 65 nucleotide sequences with a total of 734 positions in the final dataset. Evolutionary analyses were conducted in Mega 7.0 software.

Based on the canonical gene marker A56R (viral hemagglutinin) [81,82] phylogenetic analysis showed the clustering of Br-VACV isolates in two distinct clades (Figure 3) corroborating previous studies [15,17,18,33]. Following naming conventions already proposed in the literature these Br-VACV clades have been designated as Group 1, comprised of S2V, CTGV, Guarani P2 virus (GP2V), Passatempo virus (PSTV) and other non-virulent strains; and Group 2, comprised of Guarani P1 virus (GP1V), Serro virus 2011 (SH2V), Pelotas 1 virus (P1V) and other virulent strains.

Smallpox eradication remains one of the most important achievements in science and public health and, during that time, many different VACV strains were used as vaccines around the world. Consequently, its escape to the field is a plausible event. However, the presence of two biological and genetically divergent groups suggests a distinct evolutionary history for Br-VACV. The lack of complete VACV genome sequences derived from naturally circulating VACV isolates means that we can only speculate on the origins of Br-VACV strains and their relationship to vaccine strains. Despite the fact that other studies using gene markers for phylogenetic inference have consistently confirmed the two-type population structure of Br-VACV isolates, our analysis (Figure 3) only represents a small portion of the entire genome, used here as the A56R gene has been largely used in the molecular characterization of most VACV isolates during BV outbreaks [68,75,83]. An alternative hypothesis worth considering is the possible misdiagnosis of cowpox virus in Brazil in past decades. Veterinarian textbooks describe cowpox lesions in cattle and in 1985 there was, for example, a publication reporting the detection of cowpox biologically identified as cowpox virus, but with characteristics of what we now know as bovine vaccinia [84]. This could indicate the circulation of zoonotic Br-VAVC well before the Br-VAVC outbreaks that emerged during the late 1990s [85,86,87].

Our data reinforce the previous reports that indicate the circulation of two distinct Br-VACV groups. These data support the notion that both scenarios, that is, the emergence of wild isolates and vaccine escape, may have contributed to the emergence of the two distinct zoonotic Br-VACV groups in circulation. Lastly, looking beyond the intricate evolutionary history of Br-VACV, it is reasonable to be concerned that the emergence of new genetic variants could give rise to more virulent strains in the future, thereby having a greater impact on public health and in the environment.

## 3. The Bad: The Economic and Public Health Burden Associated with Bovine Vaccinia and the Alternative Routes of Zoonotic VACV Transmission in Brazil

### 3.1. The Burden of Bovine Vaccinia for Agricultural Industry in Brazil

The agricultural industry is extremely important to the Brazilian economy. Brazil is ranked as the fourth largest dairy producer in the world, and around 35 billion liters of milk were produced in 2015, generating 30 billion US dollars, and employing 4 million people [88,89]. Minas Gerais state is traditionally known for the production of milk and milk-derived products and accounts for 25.5% of the total volume of milk produced in Brazil, leading the national average [88]. As BV outbreaks mainly affect dairy cattle and milkers, the disease has a great impact on the dairy economy. Because infected individuals experience a 21-day period of illness, they became temporarily unavailable to work due to the painful vesiculopustular lesions developed, and also further systemic symptoms (Figure 2). Hence, with the rural workers sick and temporarily away from work, there is a need to acquire new dairy professionals [15,34,38,40,43,53,90,91]. Importantly, during the onset of illness, the interaction between humans and animals favors the spread of infection to the cattle herd, as well as the introduction of sick cattle to naïve herds through trade between farms or through sick dairy workers who often work at more than one property per day [36].

Regarding dairy cattle, the presence of painful vesiculopustular lesions, followed by secondary bacterial infections (Figure 2), makes the milking process difficult, resulting in a decreased milk production. The attack rate in lactating cows is generally very high, ranging from 80–100%, which can lead to a rapid viral dissemination throughout the cattle herd, also affecting calves that feed on sick lactating cows [34,53]. The vesiculopustular lesions present on the cows’ teats are very painful, and the action of milking action can lead to the loss of teats thereby reducing milk production. There is currently no evidence that milk contaminated with VACV particles can cause disease, that is, lesions on the mouth. Furthermore, there are no studies demonstrating the presence of anti-VACV antibodies in people who ingested raw milk and cheese but did not handle infected dairy cattle [92,93]. As a consequence, the small properties that depend exclusively on the dairy economy as the main source of income are largely affected. Furthermore, there are high financial costs associated with medical and veterinary expenses during the quarantine period, which is mainly a burden to small farmers [34,38,53].

Another important aspect associated with BV burden is the under-reporting of most outbreaks, and probably the absence of a differential diagnosis that could promptly improve surveillance efforts [43,92,94]. BV can easily be confused with other vesicular diseases that affect cattle, such as foot-and-mouth disease, vesicular stomatitis, pseudocowpox, bovine papular stomatitis virus (BPSV) and herpetic mamilitis [45,76,95]. Control measures can be implemented to reduce and prevent the spread of VACV, such as the suspension of trade and/or traffic of the dairy herd, as some authors have demonstrated the spread of VACV through cattle movement [96,97].

### 3.2. Bovine Vaccinia: A Neglected Public Health Concern

In natural VACV infections, the classical form of transmission is through direct contact between rural workers (milkers and farmers) and infected bovines, making BV an occupational zoonosis [34,78,91]. During the milking process, rural workers that handle infected dairy cows without adequate personal protective equipment (i.e., gloves) can easily be infected with VACV. Infected individuals commonly develop vesicular lesions on their hands and forearms, and, as VACV is epitheliotropic, the infection can easily disseminate throughout the body by self-inoculation [40,53,98].

BV outbreaks have high morbidity, with active infections reported in approximately 80% of humans working on affected properties and has been attributed to immune modulation by VACV [99]. Despite the impact of BV outbreaks on the dairy economy and public health, epidemiological surveillance is not sufficient to monitor and control the disease, and the number of human cases is still underestimated [92]. The incidence and prevalence of VACV infections are poorly studied in Brazil. Recently, Costa and colleagues detected neutralizing antibodies against OPV at a prevalence of 30.8% in a rural population from an important dairy basin in Minas Gerais state, where several BV outbreaks have been recorded [43,68,93]. Although 32.1% of individuals in that study were vaccinated against smallpox, almost 20% of non-vaccinated individuals were exposed to zoonotic VACV infections [92]. Several risk factors for VACV infection were noted in the study group including being employed as rural workers, direct contact with bovines and equids, milking, contact with raw milk for cheese production, and the occurrence of previous BV outbreak in the area [92]. Likewise, Mota et al. observed an overall seroprevalence of 27.9% in individuals from Amazonian rural villages, with 23.4% of non-vaccinated individuals exposed to zoonotic VACV infections.

Another important concern associated with BV outbreaks is the inadequate treatment of infected individuals, related to the difficulty of healthcare professionals in recognizing the disease [34,43,94]. Clinically, human VACV infection can be confused with other similar vesiculopustular infections such as parapoxviruses, leishmaniosis, mycosis, staphylococcal or *Bacillus anthracis*, making an accurate diagnosis difficult [54,94,100,101]. Concerning the similarity between the infections caused by parapoxvirus and orthopoxvirus, it is important to highlight that cases of co-circulation and co-infection between parapoxvirus and VACV have already been described in Brazil, making the possibility of a clinical diagnosis even more difficult [54,94,100,101].

A recent study evaluated the knowledge of healthcare professionals from a BV endemic area, and confirmed that 43.1% of participants were unaware of BV and aspects related to zoonotic VACV infections [94]. A common theme in the literature is that person-to-person VACV transmission can easily occur due to the direct contact with individuals recently vaccinated against smallpox [102,103,104,105,106,107], and direct contact with individuals naturally infected with zoonotic VACV [93,104,108]. Hence, person-to-person transmission should not be neglected during zoonotic BV outbreaks as infected individuals could act as possible sources of infection for healthcare professionals, increasing the burden to public health [94]. Furthermore, the possibility of nosocomial VACV spread should not be neglected [109], and further attention should be given to these infections, mainly due to the possible fatal complications that can occur in immunocompromised patients [102,110].

## 4. Alternative Routes of Zoonotic VACV Infections

As already mentioned, BV outbreaks and VACV infections have been described in all Brazilian territories (Figure 1, Supplementary Table S1), and most affected hosts are humans and bovines [34,91]. However, many other hosts have been suggested to participate in the VACV transmission chain [18,34,69,70,74,91], as well as additional forms of zoonotic VACV transmission [93]. Since the detection of anti-OPV neutralizing antibodies in residents of rural settlements in the Brazilian Amazon (Acre state), a region without any reports of BV outbreaks previously, some authors have discussed other possible VACV exposure routes to humans [111]. No correlation was observed between antibody detection and contact with cattle in this region. These data point towards alternative routes of OPV exposure and the hypothesis proposed is the close interaction between these individuals and the wild environment [111]. However, Costa and colleagues, upon evaluating a rural population from a BV endemic area, did not observe a correlation between anti-OPV neutralizing antibodies and contact with wild environments. Despite this, access to wild areas and contact with wild animals should be better explored as a possible route of human infection [92].

Corroborating the hypothesis that alternative routes of VACV transmission exist in Brazil, a family cluster was investigated for a possible case of person-to-person VACV infection [93]. Neutralizing antibodies and VACV DNA were detected in the blood of subjects, and manual milking was excluded as the main source of exposure. It was assumed that transmission had occurred via direct contact between the test subject and the farmer who had VACV lesions on his hands [93]. Previous studies of intrafamilial transmission were described in which VACV was transmitted by milkers to other residents on their properties through direct contact [104,108]. In addition, VACV was isolated from domestic utensils in the home environment of an infected patient during a BV outbreak [58]. Indeed, VACV particles are resistant in the environment, remaining viable across a range of temperatures or associated with organic matter [112,113]. However, in the family cluster study mentioned previously, participants reported the usual consumption of raw milk and artisanal cheese [93]. Taking this into account, the possible role of milk and dairy products as a source of infection has raised interesting questions regarding VACV epidemiological cycle [50,76,114,115]. Some reports support the hypothesis that milk is a potential source of VACV exposure and/or transmission. The first evidence in support of this was the isolation of VACV from milk samples during BV outbreaks in Minas Gerais [76]. Viral particles could remain viable even after contaminated milk being submitted to different thermal treatments [114]. Recently, Rehfeld and colleagues evaluated the ripening process applied to reduce cheese contamination and modify its physical and chemical characteristics. However, VACV infectious particles persisted throughout and following the ripening process and were isolated 60 days after the ripening period. Additionally, VACV DNA was detected in milk from symptomatic and asymptomatic dairy cows, including properties where BV outbreaks had not reported [50].

**Figure 4:**
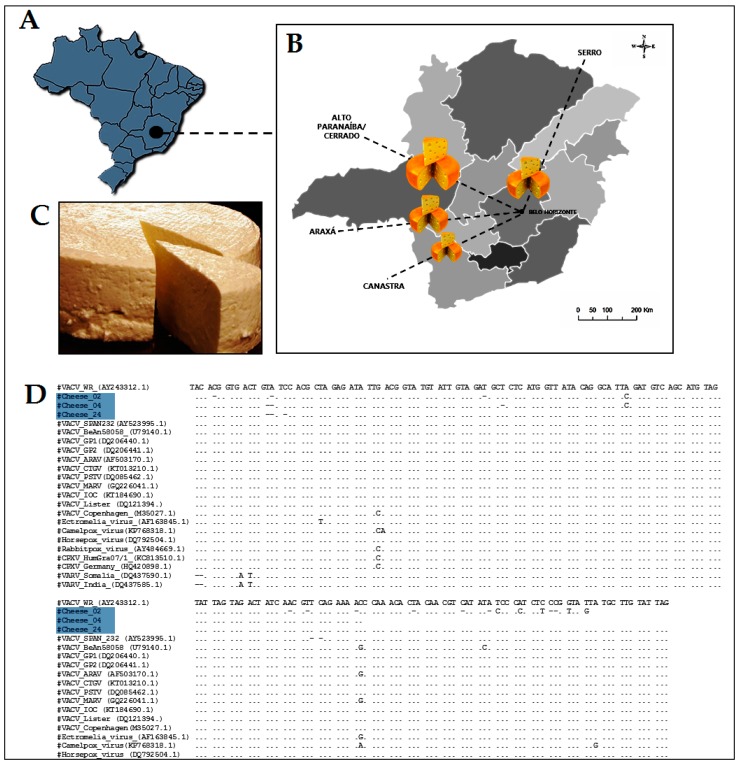
Distribution of artisanal cheese samples in four different dairy basin in Minas Gerais state. (**A**) Map of Brazil highlighting where Minas Gerais state and Belo Horizonte city are located; (**B**) All samples were collected in Belo Horizonte city, in the central area of Minas Gerais; (**C**) An example of artisanal cheese produced in Minas Gerais state; (**D**) Nucleotide sequence of the VACV detected in commercial artisanal cheese samples (blue) C11R (viral growth factor) gene compared with homologous sequences of several other orthopoxviruses. The amplified fragments were sequenced in both orientations by the dideoxy method in an ABI3130 platform (Applied Biosystems, Foster City, CA, USA), and sequence quality was analyzed by using Sequence Scanner Software 1.0 (Applied Biosystems, Foster City, CA, USA). Sequences were aligned (ClustalW (http://www.genome.jp/tools/clustalw). Information regarding chesse samples processing has been included as Supplementary Material.

In Minas Gerais state, artisanal cheese has been recognized as an intangible heritage item, and is traditionally made using raw milk. To better understand the role of artisanal cheese as a possible source of VACV infection, we analyzed commercial artisanal cheese samples produced in Minas Gerais State. A total of 38 samples were collected from June 2015 to June 2017 in Belo Horizonte city, however, the samples were produced on dairy basins, corresponding to the following cities or state regions: Serro, Araxá, Alto Paranaíba/ Cerrado and Canastra (Figure 4).

OPV-specific nested-PCR was performed targeting the C11R gene. Real-time PCR targeting A56R gene was also performed [51,80,116]. Eight samples (21.0%) tested positive for C11R gene, and three (7.9%) for A56R (Table 1). Only one sample (2.6%) tested positive for both targets. The alignment of the C11R fragments showed high similarity to the other VACV isolates from Brazil (Figure 4).

To our knowledge, this is the first report on the detection of VACV DNA in commercial artisanal cheeses in Brazil. Previous studies also analyzed viral viability in experimentally contaminated milk and its derivatives [50,76,114]. The consumption of artisanal cheese has already been identified as a possible new route of VACV transmission [92,93]. This relationship was also discussed during a buffalopox virus outbreak in India [98]. However, it was not clear in that study whether oral lesions resulted from milk consumption or the autoinoculation process [91]. Furthermore, Rehfeld and colleagues presented data on the transmission of VACV through the ingestion of contaminated milk using a mouse model. In addition, systemic viral spread with molecular detection of VACV DNA in the oral mucosa and feces was observed, even though all animals remained asymptomatic [117].

A study by de Oliveira et al. has demonstrated that VACV is not inactivated after thermal treatment [114]. Combined with the results presented here we believe that viral particles remaining viable and infectious throughout the ripening process is plausible and the consumption of artisanal cheeses may represent a real route of human exposure to the virus. It should be emphasized that the artisanal cheeses produced in the Minas Gerais are commercialized all over Brazil and other countries. No clinical cases of VACV infection have been directly linked to the consumption of milk or milk-derived products despite their widespread consumption in Brazil and elsewhere. Consequently, additional studies are necessary to clarify the role of raw milk and its derivatives in the VACV transmission chain.

## 5. The Ugly: Spreading of VACV to Urban Environments

The emergence of VACV in Brazil has had a significant impact on both the dairy economy and public health. Moreover, recent findings related VACV circulation in urban areas have raised a greater concern due to the risks and burden that could be associated with human infections.

Dutra and collaborators recently detected VACV DNA in capybaras (*Hydrochoerus hydrochaeris*) in wild areas in Pantanal and Minas Gerais state, but also in urban areas of Belo Horizonte city, Minas Gerais [69]. Capybaras are the largest wild rodent in the world, restricted to the Americas, adapt easily to anthropic environments, and can easily transit between rural and urban environments [74,118]. Hence, some investigators have suggested that these animals could transfer VACV between rural and domestic environments as they transit between farms and urban areas.

Another study describes the detection of VACV in domestic cats from urban areas in Brazil [70]. OPV neutralizing antibodies and VACV DNA were detected in domestic cats from Belo Horizonte city, Minas Gerais state [65]. Two important aspects of this study should be highlighted in the context of VACV ecology: (1) the geographic areas in which VACV-positive cats were detected comprise areas with little verticalization and the presence of green areas containing domestic animals such as bovines and equids, and wild animals (rodents, coatis, etc.); (2) the highest number of VACV-positive cats were detected in the region of Pampulha, the same region in which we had demonstrated VACV circulation in capybaras [69]. These data demonstrate that the virus is circulating in the urban environment and that the detection of VACV in capybaras in this area was not an isolated phenomenon. Considering the presence of bovines, equids and also wild rodents in this region, we may be facing the establishment of a new and still poorly understood urban cycle of VACV transmission [65]. Also, these findings highlight the possible threat this situation poses to non-vaccinated individuals and to public health in the region. If we consider the absence of vaccination and the lack of specific treatments together with the high population contingent that can be exposed to the virus, the circulation of VACV in urban environments certainly poses an ugly scenario. Also, another aspect regarding VACV emergence in large human populations that should be explored in further studies is the potential for increased virulence, associated with human adaptation and host range changes, as has been demonstrated for the variola virus and recently for monkeypox virus [2,119].

**Figure 5:**
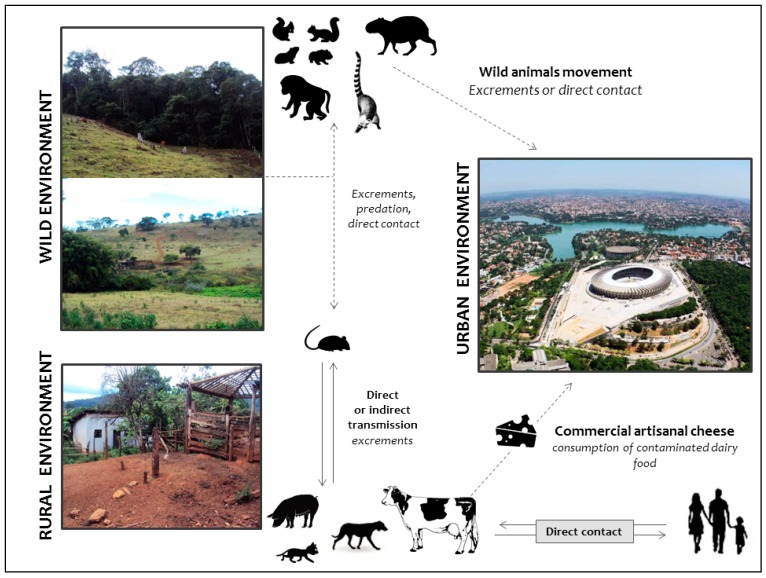
Hypothetical model highlighting the dynamic of vaccinia virus circulation in different hosts from wild, rural and urban environments. VACV outbreaks have been largely described in rural areas, affecting mainly dairy cattle and milkers. Equids have also been affected, although there are no human cases associated with direct contact with horses. Peridomestic rodents have been postulated as the link between bovine vaccinia (BV) outbreaks in dairy farms and VACV circulation in wildlife. Wild rodents could act as VACV reservoirs and transmit the virus to other small mammals, as well as peridomestic rodents, thus maintaining the wild-rural cycle. In urban areas, the dynamic also involves wild rodents that could be in contact with other mammals such as capybaras and coatis. These wild species can interact with domestic animals such as dogs and cats that live in regions bordering green areas (natural parks and forest reserves), which could favor VACV spread and transmission to other domestic animals and humans. Alternatively, VACV could be disseminated to urban areas through contaminated dairy products. Solid lines represent hypotheses already described. Dashed lines indicate new hypotheses pointed out by our group.

We propose a hypothetical model based on a previous study by Abrahão et al. [56]. Based on current trends, this hypothetical model could illustrate the dynamic of VACV circulation in urban, rural and wild areas (Figure 5), and also considers important information regarding the role of domestic animals and wildlife in the VACV natural cycle. Initially, VACV outbreaks were described in rural environments affecting dairy cattle and humans. Later, it was demonstrated that other farming animals such as equids could be implicated in the VACV transmission chain. No VACV transmission has been described from equids to humans until the recently. It has been hypothesized that wild rodents could be VACV reservoirs, and peridomestic rodents could act as the link for VACV spread between wild and rural environments, promoting the transmission among wild mammals and dairy cattle, and other farm animals and humans. The circulation of VACV in wild animals such as capybaras and coatis in urban areas could favor the direct spread of VACV between wild and urban environments. Another alternative route of VACV spread in urban areas is through contaminated commercial artisanal cheese. However, while VACV could be detected in dairy food, this alternative route is still poorly understood and further studies are necessary to clarify the role of dairy products in the transmission of VACV.

## 6. Concluding Remarks

Twenty years have passed since the first report confirming the circulation of vaccinia virus in Brazil. At that time, the researchers involved in the isolation and characterization of these wild and/or zoonotic isolates found it difficult to convince the scientific community that these isolates were real sylvan isolates and not mere laboratory contaminants. Today, however, the circulation of VACV in wild, rural and even urban environments in Brazil is a scientific fact. In response, a new line of discussion is necessary, at the very center of which lies an important question: how should public health authorities respond to VACV circulation to protect public health interests?

This review seeks to establish what we consider the positive and negative aspects related to the emergence of VACV in Brazil. We highlight the possibility of broadening our understanding about the ecological and evolutionary aspects of the virus as a positive aspect of VACV emergence in the region (the good), though we also discuss the negative impact that bovine vaccinia virus has had on the milk economy and on public health (the bad). This situation becomes ugly when we consider the evidence for VACV circulation in urban environments, which could have extremely dire consequences as the virus encounters a large non-immune population.

In Brazil, as in most of the world, vaccination against smallpox was terminated in the 1970s and the last case of smallpox in the country was described in April 1971. Therefore, a large part of the population is no longer immune to poxvirus infections, either because immunity has waned over the last 40 years or simply because the majority of people are too young to have been vaccinated. As of today, smallpox vaccines, composed of less virulent strains of vaccinia virus such as the Modified vaccinia virus Ankara (MVA) or the ACAM2000™—a plaque-purified derivative of the US Dryvax Vaccine—are available in many countries as a result of the heightened fear of smallpox reintroduction [10,120,121]. Nonetheless, would it be feasible to vaccinate Brazilians against vaccinia virus? From an immunological point of view, there are few doubts about the efficacy of smallpox vaccines against those VACV isolates (although no studies on the matter have been systematically conducted). Thus the real question is whether it would truly be necessary. This question is far more difficult to answer. At this point, there is an unequivocal pattern of virus spread in the country, as a growing number of isolates have been obtained all over Brazil, from different hosts, and in different environments (Figure 1). Likewise, increased contact between these viruses and people has been documented [58,93,108]. Furthermore, many would say that the difficulties—or even risks—posed by a mass vaccination campaign using poxvirus vaccines in people is simply not justifiable in terms of the number of individuals at risk of infection. Indeed, due to the fact that the notification of BV is not compulsory in the country, we do not have access to the real number of cases in humans. Besides, we also do not have access to official data regarding adverse effects that arise from smallpox vaccination in the country, which prevents us from considering the real benefit of resuming vaccination. Others may say that the availability of new anti-poxvirus drugs, such as the ST-246 and CMX-001 [121,122], could circumvent the constraints of mass vaccination against orthopoxviruses as the number of affected people has not yet been considered indicative of an epidemic. Moreover, vaccinia immunoglobulin therapies also represent a feasible approach to treatment or prophylaxis that could undermine the need of mass vaccination [123]. However, even if we have drugs and immunoglobulin therapies available to treat VACV infections, Brazil still has issues related to treatment costs and implementation policies.

While this picture is alarming, the occurrence of BV in Brazil still represents a big question mark from a public health point of view, as scientists and officials alike are uncertain of the true public health significance of this disease and, consequently, how to manage it.

## Figures and Tables

**Table 1:** Commercial artisanal cheese samples produced in four different dairy basins in Minas Gerais state tested for the presence of vaccinia virus DNA, 2015–2017.

Sample	Date (Month/Year)	Dairy Basin	Nested-PCR C11R	Real-Time PCR A56R
01	June/2015	Serro	+	−
02	June/2015	Serro	+	−
03	June/2015	Serro	−	−
04	June/2015	Serro	+	−
05	June/2015	Serro	−	−
06	June/2015	Serro	+	−
07	February/2016	Serro	+	−
08	March/2016	Serro	−	−
09	April/2016	Serro	−	−
10	April/2016	Serro	−	−
11	April/2016	Alto do Paranaíba/Cerrado	+	+
12	May/2016	Araxá	+	−
13	August/2016	Araxá	−	+
14	September/2016	Araxá	−	−
15	September/2016	Araxá	−	+
16	September/2016	Alto do Paranaíba/Cerrado	−	−
17	October/2016	Araxá	−	−
18	March/2017	Serro	−	−
19	March/2017	Alto do Paranaíba/Cerrado	−	−
20	March/2017	Araxá	−	−
21	April/2017	Araxá	−	−
22	April/2017	Serro	−	−
23	April/2017	Serro	−	−
24	April/2017	Serro	+	−
25	May/2017	Araxá	−	−
26	May/2017	Serro	−	−
27	May/2017	Alto do Paranaíba/Cerrado	−	−
28	May/2017	Serro	−	−
29	May/2017	Canastra	−	−
30	June/2017	Araxá	−	−
31	June/2017	Serro	−	−
32	June/2017	Serro	−	−
33	June/2017	Araxá	−	−
34	June/2017	Serro	−	−
35	June/2017	Serro	−	−
36	June/2017	Araxá	−	−
37	June/2017	Serro	−	−
38	June/2017	Araxá	−	−

+: Positive samples; −: negative samples.

## Supplementary Materials

The following are available online at www.mdpi.com/1999-4915/9/11/340/s1.

## References

[1] Damon I. (2013). Poxviruses.

[2] Duggan A.T., Perdomo M.F., Piombino-Mascali D., Marciniak S., Poinar D., Emery M.V., Buchmann J.P., Duchêne S., Jankauskas R., Humphreys M. (2016). 17th century variola virus reveals the recent history of smallpox. Curr. Biol..

[3] Fenner F., Henderson D. (1988). Smallpox and Its Eradication.

[4] Henderson D., Preston R. (2009). Smallpox: The Death of a Disease—The Inside Story of Eradicating a Worldwide Killer.

[5] Thèves C., Biagini P., Crubézy E. (2014). The rediscovery of smallpox. Clin. Microbiol. Infect..

[6] Shchelkunov S.N. (2011). Emergence and reemergence of smallpox: The need for development of a new generation smallpox vaccine. Vaccine.

[7] Henderson D.A., Klepac P. (2013). Lessons from the eradication of smallpox: An interview with D.A. Henderson. Philos. Trans. R. Soc. Lond. B Biol. Sci..

[8] Shchelkunov S.N. (2013). An increasing danger of zoonotic orthopoxvirus infections. PLoS Pathog..

[9] Baxby D. (1981). Jenner’s Smallpox Vaccine.

[10] Sánchez-Sampedro L., Perdiguero B., Mejias-Perez E., Garcia-Arriaza J., di Pilato M., Esteban M. (2015). The evolution of poxvirus vaccines. Viruses.

[11] Downie A.W. (1939). A Study of the Lesions Produced Experimentally by Cowpox Virus.

[12] Downie A.W. (1939). The Immunological Relationship of the Virus of Spontaneous Cowpox to Vaccinia Virus.

[13] Damaso C.R. (2017). Revisiting Jenner’s mysteries, the role of the Beaugency lymph in the evolutionary path of ancient smallpox vaccines. Lancet Infect. Dis..

[14] World Health Organization (WHO) (1982). Archives of the Smallpox Eradication Programme.

[15] Trindade G.S., Emerson G.L., Carroll D.S., Kroon E.G., Damon I.K. (2007). Brazilian vaccinia viruses and their origins. Emerg. Infect. Dis..

[16] Moussatché N., Damaso C.R., McFadden G. (2008). When good vaccines go wild: Feral Orthopoxvirus in developing countries and beyond. J. Infect. Dev. Ctries.

[17] Medaglia M.L., Moussatché N., Nitsche A., Dabrowski P.W., Li Y., Damon I.K., Lucas C.G., Arruda L.B., Damaso C.R. (2015). Genomic analysis, phenotype, and virulence of the historical brazilian smallpox vaccine strain IOC: Implications for the origins and evolutionary relationships of vaccinia virus. J. Virol..

[18] Miranda J.B., Borges I.A., Campos S.P., Vieira F.N., de Ázara T.M., Marques F.A., Costa G.B., Luis A.P.M., de Oliveira J.S., Ferreira P.C.P. (2017). Serologic and molecular evidence of vaccinia virus circulation among small mammals from different biomes, Brazil. Emerg. Infect. Dis..

[19] Venkatesan G., Balamurugan V., Prabhu M., Yogisharadhya R., Bora D.P., Gandhale P.N., Sankar M.S., Kulkarni A.M., Singh R.K., Bhanuprakash V. (2010). Emerging and re-emerging zoonotic buffalopox infection: A severe outbreak in Kolhapur (Maharashtra), India. Vet. Ital..

[20] Singh R.K., Balamurugan V., Bhanuprakash V., Venkatesan G., Hosamani M. (2012). Emergence and reemergence of vaccinia-like viruses: Global scenario and perspectives. Indian J. Virol..

[21] Goyal T., Varshney A., Bakshi S.K., Barua S., Bera B.C., Singh R.K. (2013). Buffalo pox outbreak with atypical features: A word of caution and need for early intervention!. Int. J. Dermatol..

[22] Baxby D., Hill B.J. (1971). Characteristics of a new poxvirus isolated from Indian buffaloes. Arch. Gesamte Virusforsch..

[23] Chandra R., Singh I.P., Garg S.K., Varshney K.C. (1986). Experimental pathogenesis of buffalo pox virus in rabbits: Clinico-pathological studies. Acta Virol..

[24] Dumbell K., Richardson M. (1993). Virological investigations of specimens from buffaloes affected by buffalopox in Maharashtra State, India between 1985 and 1987. Arch. Virol..

[25] Nedunchelliyan S., Reddy D.S., Venkataraman K.S. (1992). Buffalo pox infection in man. Indian J. Public Health.

[26] Kolhapure R.M., Deolankar R.P., Tupe C.D., Raut C.G., Basu A., Dama B.M., Pawar S.D., Joshi M.V., Padbidri V.S., Goverdhan M.K. (1997). Investigation of buffalopox outbreaks in Maharashtra State during 1992–1996. Indian J. Med. Res..

[27] Bhanuprakash V., Venkatesan G., Balamurugan V., Hosamani M., Yogisharadhya R., Chauhan R.S., Pande A., Mondal B., Singh R.K. (2010). Pox outbreaks in sheep and goats at Makhdoom (Uttar Pradesh), India: Evidence of sheeppox virus infection in goats. Transbound. Emerg. Dis..

[28] Singh R.K., Hosamani M., Balamurugan V., Satheesh C.C., Shingal K.R., Tatwarti S.B., Bambal R.G., Ramteke V., Yadav M.P. (2006). An outbreak of buffalopox in buffalo (Bubalus bubalis) dairy herds in Aurangabad, India. Rev. Sci. Tech..

[29] Fenner F. (1958). The biological characters of several strains of vaccinia, cowpox and rabbitpox viruses. Virology.

[30] DiGiacomo R.F., Maré C. (1994). The Biology of the Laboratory Rabbit.

[31] Franco-Luiz A.P., Fagundes-Pereira A., Costa G.B., Alves P.A., Oliveira D.B., Bonjardim C.A., Ferreira P.C.P., de Souza Trindade G., Panei C.J., Galosi C.M. (2014). Spread of vaccinia virus to cattle herds, Argentina, 2011. Emerg. Infect. Dis..

[32] Franco-Luiz A.P., Oliveira D.B., Pereira A.F., Gasparini M.C.S., Bonjardim C.A., Ferreira P.C.P., de Souza Trindade G., Puentes R., Furtado A., Abrahão J.S. (2016). Detection of vaccinia virus in dairy cattle serum samples from 2009, uruguay. Emerg. Infect. Dis..

[33] Usme-Ciro J.A., Paredes A., Walteros D.M., Tolosa-Pérez E.N., Laiton-Donato K., del Carmen Pinzón M., Petersen B.W., Gallardo-Romero N.F., Li Y., Wilkins K. (2017). Detection and molecular characterization of zoonotic poxviruses circulating in the amazon region of Colombia, 2014. Emerg. Infect. Dis..

[34] Kroon E.G., Mota B.E.F., Abrahão J.S., da Fonseca F.G., de Souza Trindade G. (2011). Zoonotic Brazilian Vaccinia virus: From field to therapy. Antivir. Res..

[35] Damaso C.R., Esposito J.J., Condit R.C., Moussatché N. (2000). An emergent poxvirus from humans and cattle in Rio de Janeiro State: Cantagalo virus may derive from Brazilian smallpox vaccine. Virology.

[36] De Souza Trindade G., da Fonseca F.G., Marques J.T., Nogueira M.L., Mendes L.C.N., Borges A.S., Peiró J.R., Pituco E.M., Bonjardim C.A., Ferreira P.C.P. (2003). Araçatuba virus: A vaccinialike virus associated with infection in humans and cattle. Emerg. Infect. Dis..

[37] Nagasse-Sugahara T.K., Kisielius J.J., Ueda-Ito M., Curti S.P., Figueiredo C.A., Cruz Á.S., Silva M.M.J., Ramos C.H., Silva M.C.C., Sakurai T. (2004). Human vaccinia-like virus outbreaks in São Paulo and Goiás States, Brazil: Virus detection, isolation and identification. Rev. Inst. Med. Trop. Sao Paulo.

[38] Leite J.A., Drumond B.P., Trindade G.S., Lobato Z.I., da Fonseca F.G., dos Santos J.R., Madureira M.C., Guedes M.I., Ferreira J.M., Bonjardim C.A. (2005). Passatempo virus, a vaccinia virus strain, Brazil. Emerg. Infect. Dis..

[39] De Souza Trindade G., Drumond B.P., Guedes M.I.M.C., Leite J.A., Mota B.E.F., Campos M.A., da Fonseca F.G., Nogueira M.L., Lobato Z.I.P., Bonjardim C.A. (2007). Zoonotic vaccinia virus infection in Brazil: Clinical description and implications for health professionals. J. Clin. Microbiol..

[40] Silva L., Angerami R. (2008). Viroses Emergentes No Brasil.

[41] Megid J., Appolinário C.M., Langoni H., Pituco E.M., Okuda L.H. (2008). Vaccinia virus in humans and cattle in southwest region of Sao Paulo state, Brazil. Am. J. Trop. Med. Hyg..

[42] Silva-Fernandes A.T., Travassos C.E.P.F., Ferreira J.M.S., Abrahão J.S., de Oliveira Rocha E.S., Viana-Ferreira F., dos Santos J.R., Bonjardim C.A., Ferreira P.C.P., Kroon E.G. (2009). Natural human infections with Vaccinia virus during bovine vaccinia outbreaks. J. Clin. Virol..

[43] Trindade G.S., Guedes M.I., Drumond B.P., Mota B.E., Abrahão J.S., Lobato Z.I., Gomes J.A., Corrêa-Oliveira R., Nogueira M.L., Kroon E.G. (2009). Zoonotic vaccinia virus: Clinical and immunological characteristics in a naturally infected patient. Clin. Infect. Dis..

[44] Megid J., Borges I.A., Abrahão J.S., Trindade G.S., Appolinário C.M., Ribeiro M.G., Allendorf S.D., Antunes J.M.A., Silva-Fernandes A.T., Kroon E.G. (2012). Vaccinia virus zoonotic infection, São Paulo State, Brazil. Emerg. Infect. Dis..

[45] Sant’ana F.F., Leal A.D.A., Rabelo R.E., Vulcani V.A., Junior F., Jair A., Cargnelutti J.F., Flores E.F. (2013). Outbreaks of vesicular diseases caused by Vaccinia virus in dairy cattle from Goiás State, Brazil (2010–2012). Pesqui. Vet. Bras..

[46] Schatzmayr G.H., Romijn P.C., Barreto D.F., Silva E.E., da Costa Farias Filho J., Tavares A.F.D.A., Barth O.M. (2005). An outbreak of vesicopustular disease in humans and dairy cattle in the state of Rio de Janeiro in 2006. Virus Rev. Res..

[47] Rivetti A.V., Guedes M.I.M., Rehfeld I.S., Oliveira T.M., Matos A.C.D., Abrahão J.S., Kroon E.G., Lobato Z.I. (2013). Bovine vaccinia, a systemic infection: Evidence of fecal shedding, viremia and detection in lymphoid organs. Vet. Microbiol..

[48] Guedes M.I., Rehfeld I.S., Oliveira T.M.L., Assis F.L., Matos A.C.D., Abrahão J.S., Kroon E.G., Lobato Z.I.P. (2013). Detection of Vaccinia virus in blood and faeces of experimentally infected cows. Transbound. Emerg. Dis..

[49] Rehfeld I.S., Guedes M.I.M., Matos A.C.D., de Oliveira T.M., Junior A.V.R., Moura A.C.J., Paes P.R.O., do Lago L.A., Kroon E.G., Lobato Z.I.P. (2013). Clinical, hematological and biochemical parameters of dairy cows experimentally infected with Vaccinia virus. Res. Vet. Sci..

[50] Rehfeld I.S., Fraiha A.L.S., Matos A.C.D., Guedes M.I.M., Costa E.A., de Souza M.R., Cavalcante L.F., Lobato Z.I. (2017). Short communication: Survival of Vaccinia virus in inoculated cheeses during 60-day ripening. J. Dairy Sci..

[51] De Souza Trindade G., Li Y., Olson V.A., Emerson G., Regnery R.L., da Fonseca F.G., Kroon E.G., Damon I. (2008). Real-time PCR assay to identify variants of Vaccinia virus: Implications for the diagnosis of bovine vaccinia in Brazil. J. Virol. Methods.

[52] Rehfeld I.S., Matos A.C.D., Guedes M.I.M.C., Costa A.G., Fraiha A.L.S., Lobato Z.I.P. (2017). Subclinical bovine vaccinia: An important risk factor in the epidemiology of this zoonosis in cattle. Res. Vet. Sci..

[53] Lobato Z., Trindade G.S., Frois M.C.M., Ribeiro E.B.T., Dias G.R.C., Teixeira B.M., Lima F.A., Almeida G.M.F., Kroon E.G. (2005). Outbreak of exantemal disease caused by Vaccinia virus in human and cattle in Zona da Mata region, Minas Gerais. Arq. Bras. Med. Vet. Zootec..

[54] Abrahão J.S., Silva-Fernandes A.T., Assis F.L., Guedes M.I., Drumond B.P., Leite J.A., Coelho L.F., Turrini F., Fonseca F.G., Lobato Z.I. (2010). Human Vaccinia virus and Pseudocowpox virus co-infection: Clinical description and phylogenetic characterization. J. Clin. Virol..

[55] Trindade G., Guedes M.I.M.C., Costa G.B., Figueiredo P.O., Abrahão J.S., Kroon E.G., Foseca F.G. (2014). A 31 year-old Brazilian man with exanthematous lesions. J. Vaccines Vaccin..

[56] Abrahão J.S., Guedes M.I.M., Trindade G.S., Fonseca F.G., Campos R.K., Mota B.F., Lobato Z.I., Silva-Fernandes A.T., Rodrigues G.O., Lima L.S. (2009). One more piece in the VACV ecological puzzle: Could peridomestic rodents be the link between wildlife and bovine vaccinia outbreaks in Brazil?. PLoS ONE.

[57] Schatzmayr H.G., Costa R.V.C., Gonçalves M.C.R., Barreto D.F., Batista V.H., Silva M.E.V., Brust L.A.C., Barth O.M. (2009). Human infections caused by vaccinia-like poxviruses in Brazil. Rev. Soc. Bras. Med. Trop..

[58] Assis F.L., Borges I.A., Mesquita V.S., Ferreira P.C., Trindade G.S., Kroon E.G., Abrahão J.S. (2013). Vaccinia virus in household environment during bovine vaccinia outbreak, Brazil. Emerg. Infect. Dis..

[59] Abrahão J.S., Campos R.K., de Souza Trindade G., da Fonseca F.G., Ferreira P.C.P., Kroon E.G. (2015). Outbreak of severe zoonotic vaccinia virus infection, Southeastern Brazil. Emerg. Infect. Dis..

[60] Oliveira D.B., Assis F.L., Ferreira P.C.P., Bonjardim C.A., de Souza Trindade G., Kroon E.G., Abrahão J.S. (2013). Group 1 Vaccinia virus zoonotic outbreak in Maranhao State, Brazil. Am. J. Trop. Med. Hyg..

[61] Franco-Luiz A.P., Pereira A.F., de Oliveira C.H.S., Barbosa J.D., Oliveira D.B., Bonjardim C.A., Ferreira P.C.P., de Souza Trindade G., Abrahão J.S., Kroon E.G. (2016). The detection of Vaccinia virus confirms the high circulation of Orthopoxvirus in buffaloes living in geographical isolation, Marajó Island, Brazilian Amazon. Comp. Immunol. Microbiol. Infect. Dis..

[62] Fonseca F.G., Lanna M.C.S., Campos M.A.S., Kitajima E.W., Peres J.N., Golgher R.R., Ferreira P.C.P., Kroon E.G. (1998). Morphological and molecular characterization of the poxvirus BeAn 58058. Arch. Virol..

[63] Da Fonseca F.G., Trindade G.S., Silva R.L., Bonjardim C.A., Ferreira P.C., Kroon E.G. (2002). Characterization of a vaccinia-like virus isolated in a Brazilian forest. J. Gen. Virol..

[64] Marques J.T., de Souza Trindade G., Da Fonseca F.G., Dos Santos J.R., Bonjardim C.A., Ferreira P.C.P., Kroon E.G. (2001). Characterization of ATI, TK and IFN-α/βR genes in the genome of the BeAn 58058 virus, a naturally attenuated wild Orthopoxvirus. Virus Genes.

[65] (2015). Brasil, Rebanho Bovino Brasileiro Cresce e Chega a 212.3 Milhões de Cabeças de Gado. http://www.brasil.gov.br/.

[66] Ministério da Agricultura, Pecuária e Abastecimento (MAPA) Dados de Rebanho Bovino e Bubalino No Brasil—2015. http://www.agricultura.gov.br/.

[67] Quiner C.A., Nakazawa Y. (2017). Ecological niche modeling to determine potential niche of Vaccinia virus: A case only study. Int. J. Health Geogr..

[68] Assis F.L., Almeida G.M., Oliveira D.B., Franco-Luiz A.P., Campos R.K., Guedes M.I., Fonseca F.G., Trindade G.S., Drumond B.P., Kroon E.G. (2012). Characterization of a new Vaccinia virus isolate reveals the C23L gene as a putative genetic marker for autochthonous Group 1 Brazilian Vaccinia virus. PLoS ONE.

[69] Peres M.G., Barros C.B., Appolinário C.M., Antunes J.M., Mioni M.S., Bacchiega T.S., Allendorf S.D., Vicente A.F., Fonseca C.R., Megid J. (2016). Dogs and opossums positive for Vaccinia virus during outbreak affecting cattle and humans, São Paulo State, Brazil. Emerg. Infect. Dis..

[70] Costa G.B., Miranda J.B., Almeida G.G., de Oliveira J.S., Pinheiro M.S., Gonçalves S.A., dos Reis J.K.P., Gonçalves R., Ferreira P.C.P., Bonjardim C.A. (2017). Detection of Vaccinia virus in urban domestic cats, Brazil. Emerg. Infect. Dis..

[71] Abrahão J.S., Souza Trindade G., Pereira-Oliveira G., Oliveira Figueiredo P., Costa G., Moreira Franco-Luiz A.P., Lopes Assis F., Bretas de Oliveira D., Mattos Paim L.R., Araújo Oliveira C.E. (2017). Detection of Vaccinia virus during an outbreak of exanthemous oral lesions in Brazilian equids. Equine Vet. J..

[72] Abrahão J.S., Silva-Fernandes A.T., Lima L.S., Campos R.K., Guedes M.I., Cota M.M., Assis F.L., Borges I.A., Souza-Júnior M.F., Lobato Z.I. (2010). Vaccinia virus infection in monkeys, Brazilian Amazon. Emerg. Infect. Dis..

[73] Barbosa A.V., Medaglia M.L.G., Soares H.S., Quixabeira-Santos J.C., Gennari S.M., Damaso C.R. (2014). Presence of neutralizing antibodies to Orthopoxvirus in capybaras (Hydrochoerus hydrochaeris) in Brazil. J. Infect. Dev. Ctries..

[74] Dutra L.A., de Freitas Almeida G.M., Oliveira G.P., Abrahão J.S., Kroon E.G., de Souza Trindade G. (2017). Molecular evidence of Orthopoxvirus DNA in capybara (Hydrochoerus hydrochaeris) stool samples. Arch. Virol..

[75] Drumond B.P., Leite J.A., da Fonseca F.G., Bonjardim C.A., Ferreira P.C.P., Kroon E.G. (2008). Brazilian Vaccinia virus strains are genetically divergent and differ from the Lister vaccine strain. Microbes Infect..

[76] Abrahão J.S., Oliveira T.M., Campos R.K., Madureira M.C., Kroon E.G., Lobato Z.I. (2009). Bovine vaccinia outbreaks: Detection and isolation of vaccinia virus in milk samples. Foodborne Pathog. Dis..

[77] Peres M.G., Bacchiega T.S., Appolinário C.M., Vicente A.F., Allendorf S.D., Antunes J.M.A.P., Moreira S.A., Legatti E., Fonseca C.R., Pituco E.M. (2013). Serological study of vaccinia virus reservoirs in areas with and without official reports of outbreaks in cattle and humans in São Paulo, Brazil. Arch. Virol..

[78] Trindade G.S., Emerson G.L., Sammons S., Frace M., Govil D., Fernandes Mota B.E., Abrahão J.S., de Assis F.L., Olsen-Rasmussen M., Goldsmith C.S. (2016). Serro 2 virus highlights the fundamental genomic and biological features of a natural Vaccinia virus infecting humans. Viruses.

[79] Campos R.K., Brum M.C., Nogueira C.E., Drumond B.P., Alves P.A., Siqueira-Lima L., Assis F.L., Trindade G.S., Bonjardim C.A., Ferreira P.C. (2011). Assessing the variability of Brazilian Vaccinia virus isolates from a horse exanthematic lesion: Coinfection with distinct viruses. Arch. Virol..

[80] Abrahão J.S., Drumond B.P., de Souza Trindade G., da Silva-Fernandes A.T., Ferreira J.M.S., Alves P.A., Campos R.K., Siqueira L., Bonjardim C.A., Ferreira P.C.P. (2010). Rapid detection of Orthopoxvirus by semi-nested PCR directly from clinical specimens: A useful alternative for routine laboratories. J. Med. Virol..

[81] Thompson J.D., Higgins D.G., Gibson T.J. (1994). Improved sensitivity of profile searches through the use of sequence weights and gap excision. Comput. Appl. Biosci..

[82] Eddy S.R. (1995). Multiple alignment using hidden Markov models. Proc. Int. Conf. Intell. Syst. Mol. Biol..

[83] Leite J.A., Drumond B.P., de Souza Trindade G., Bonjardim C.A., Ferreira P.C.P., Kroon E.G. (2007). Brazilian Vaccinia virus strains show genetic polymorphism at the ati gene. Virus Genes.

[84] Silva P., Viana F., Ribeiro S. (1985). Surto de varíola bovina no município de Prata—MG. Bras. Med. Vet. Zootec..

[85] Rodrigues-Da-Silva G., Rabello S.I., Angulo J.J. (1963). Epidemic of variola minor in a suburb of São Paulo. Public Health Rep..

[86] De Quadros C.C., Morris L., da Costa E.A., Arnt N., Tigre C.H. (1972). Epidemiology of variola minor in Brazil based on a study of 33 outbreaks. Bull. World Health Organ..

[87] Behbehani A.M. (1983). The smallpox story: Life and death of an old disease. Microbiol. Rev..

[88] (2016). Instituto Brasileiro de Geografia e Estatística (IBGE). https://www.ibge.gov.br/.

[89] (2016). Empresa Brasileira de Pesquisa Agrocpecuária (EMBRAPA). https://www.embrapa.br/.

[90] Trindade G.S., Lobato Z.I., Drumond B.P., Leite J.A., Trigueiro R.C., Guedes M.I., Da Fonseca F.G., Dos Santos J.R., Bonjardim C.A., Ferreira P.C. (2006). Short report: Isolation of two vaccinia virus strains from a single bovine vaccinia outbreak in rural area from Brazil: Implications on the emergence of zoonotic orthopoxviruses. Am. J. Trop. Med. Hyg..

[91] Da Fonseca F.G., Kroon E.G., Nogueira M.L., de Souza Trindade G. (2011). Zoonotic Vaccinia virus outbreaks in Brazil. Future Virol..

[92] Costa G.B., Augusto L.T.S., Leite J.A., Ferreira P.C.P., Bonjardim C.A., Abrahão J.S., Kroon E.G., Moreno E.C., de Souza Trindade G. (2016). Seroprevalence of Orthopoxvirus in rural Brazil: Insights into anti-OPV immunity status and its implications for emergent zoonotic OPV. Virol. J..

[93] Costa G.B., Borges I.A., Alves P.A., Miranda J.B., Luiz A.P.M., Ferreira P.C., Abrahão J.S., Moreno E.C., Kroon E.G., de Souza Trindade G. (2015). Alternative routes of zoonotic Vaccinia virus transmission, Brazil. Emerg. Infect. Dis..

[94] De Oliveira J.S., Costa G.B., Luiz A.P.M.F., Leite J.A., Bonjardim C.A., Abrahão J.S., Drumond B.P., Kroon E.G., de Souza Trindade G. (2017). Cross-sectional study involving healthcare professionals in a Vaccinia virus endemic area. Vaccine.

[95] Moojen V., Riet-Correa F., Roehe P.M., Weiblen R. (1996). Viroses Confundíveis Com Febre Aftosa.

[96] Quixabeira-Santos J.C., Medaglia M.L.G., Pescador C.A., Damaso C.R. (2011). Animal movement and establishment of vaccinia virus Cantagalo strain in Amazon biome, Brazil. Emerg. Infect. Dis..

[97] Laguardia-Nascimento M., Sales É.B., Gasparini M.R., de Souza N.M., da Silva J.A.G., Souza G.G., Carani F.R., dos Santos A.F., Rivetti Júnior A.V., Camargos M.F. (2016). Detection of multiple viral infections in cattle and buffalo with suspected vesicular disease in Brazil. J. Vet. Diagn. Investig..

[98] Gurav Y.K., Raut C.G., Yadav P.D., Tandale B.V., Sivaram A., Pore M.D., Basu A., Mourya D.T., Mishra A.C. (2011). Buffalopox outbreak in humans and animals in Western Maharashtra, India. Prev. Vet. Med..

[99] Silva Gomes J.A., de Araújo F.F., Trindade G.D.S., Quinan B.R., Drumond B.P., Ferreira J.M.S., Mota B.E.F., Nogueira M.L., Kroon E.G., Abrahão J.S. (2012). Immune modulation in primary Vaccinia virus zoonotic human infections. Clin. Dev. Immunol..

[100] Alves P.A., Figueiredo P.O., de Oliveira C.H., Barbosa J.D., Lima D.H., Bomjardim H.A., Silva N.S., Campos K.F., Oliveira C.M.C., Barbosa-Stancioli E.F. (2016). Occurrence of Pseudocowpox virus associated to Bovine viral diarrhea virus-1, Brazilian Amazon. Comp. Immunol. Microbiol. Infect. Dis..

[101] Laguardia-Nascimento M., de Oliveira A.P.F., Azevedo I.C., Júnior A.V.R., Camargos M.F., Júnior A.A.F. (2017). Spread of poxviruses in livestock in Brazil associated with cases of double and triple infection. Arch. Virol..

[102] Vora S., Damon I., Fulginiti V., Weber S.G., Kahana M., Stein S.L., Gerber S.I., Garcia-Houchins S., Lederman E., Hruby D. (2008). Severe eczema vaccinatum in a household contact of a smallpox vaccinee. Clin. Infect. Dis..

[103] Lederman E., Miramontes R., Openshaw J., Olson V.A., Karem K.L., Marcinak J., Panares R., Staggs W., Allen D., Weber S.G. (2009). Eczema vaccinatum resulting from the transmission of Vaccinia virus from a smallpox vaccinee: An investigation of potential fomites in the home environment. Vaccine.

[104] Batista V.H., Scremin J., Aguiar L.M., Schatzmayr H.G. (2009). Vulvar infection and possible human-to-human transmission of bovine poxvirus disease. Virus Rev. Res..

[105] Hughes C.M., Blythe D., Li Y., Reddy R., Jordan C., Edwards C., Adams C., Conners H., Rasa C., Wilby S. (2011). Vaccinia virus infections in martial arts gym, Maryland, USA, 2008. Emerg. Infect. Dis..

[106] Young G.E., Hidalgo C.M., Sullivan-Frohm A., Schulte C., Davis S., Kelly-Cirino C., Egan C., Wilkins K., Emerson G.L., Noyes K. (2011). Secondary and tertiary transmission of vaccinia virus from US military service member. Emerg. Infect. Dis..

[107] Wertheimer E.R., Olive D.S., Brundage J.F., Clark L.L. (2012). Contact transmission of vaccinia virus from smallpox vaccinees in the United States, 2003–2011. Vaccine.

[108] Pereira Oliveira G., Fernandes A.T.S., de Assis F.L., Alves P.A., Luiz A.P.M.F., Figueiredo L.B., de Almeida C.M.C., Travassos C.E.P.F., de Souza Trindade G., Abrahão J.S. (2014). Intrafamilial transmission of Vaccinia virus during a bovine Vaccinia outbreak in Brazil: A new insight in viral transmission chain. Am. J. Trop. Med. Hyg..

[109] Zafar A., Swanepoel R., Hewson R., Nizam M., Ahmed A., Husain A., Grobbelaar A., Bewley K., Mioulet V., Dowsett B. (2007). Nosocomial buffalopoxvirus infection, Karachi, Pakistan. Emerg. Infect. Dis..

[110] Fassbender P., Zange S., Ibrahim S., Zoeller G., Herbstreit F., Meyer H. (2016). Generalized cowpox virus infection in a patient with HIV, Germany, 2012. Emerg. Infect. Dis..

[111] Mota B.E., Trindade G.S., Diniz T.C., da Silva-Nunes M., Braga E.M., Urbano-Ferreira M., Rodrigues G.O.L., Bonjardim C.A., Ferreira P.C.P., Kroon E.G. (2010). Seroprevalence of orthopoxvirus in an Amazonian rural village, Acre, Brazil. Arch. Virol..

[112] Essbauer S., Meyer H., Porsch-Özcürümez M., Pfeffer M. (2007). Long-lasting stability of vaccinia virus (orthopoxvirus) in food and environmental samples. Zoonoses Public Health.

[113] Abrahão J.S., de Souza Trindade G., Ferreira J.M.S., Campos R.K., Bonjardim C.A., Ferreira P.C.P., Kroon E.G. (2009). Long-lasting stability of Vaccinia virus strains in murine feces: Implications for virus circulation and environmental maintenance. Arch. Virol..

[114] De Oliveira T.M., Rehfeld I.S., Siqueira J.M.F., Abrahao J.S., Campos R.K., dos Santos A.K.R., Cerqueira M.M.O., Kroon E.G., Lobato Z.I. (2010). Vaccinia virus is not inactivated after thermal treatment and cheese production using experimentally contaminated milk. Foodborne Pathog. Dis..

[115] De Oliveira T.M., Guedes M.I.M.C., Rehfeld I.S., Matos A.C.D., Rivetti A.V., Alves P.A., Galinari G.C.F., Cerqueira M.M.O.P., Abrahão J.S., Lobato Z.I.P. (2015). Detection of Vaccinia virus in milk: Evidence of a systemic and persistent infection in experimentally infected cows. Foodborne Pathog. Dis..

[116] Geessien Kroon E., Santos Abrahão J., de Souza Trindade G., Pereira Oliveira G., Luiz M.F., Paula A., Barbosa Costa G., Teixeira Lima M., Silva Calixto R., de Oliveira D.B. (2016). Natural Vaccinia virus infection: diagnosis, isolation, and characterization. Curr. Protoc. Microbiol..

[117] Rehfeld I.S., Guedes M.I.M.C., Fraiha A.L.S., Costa A.G., Matos A.C.D., Fiúza A.T.L., Lobato Z.I.P. (2015). Vaccinia virus transmission through experimentally contaminated milk using a murine model. PLoS ONE.

[118] Ferraz K.M.P.M.B., Manly B., Verdade L.M. (2010). The influence of environmental variables on capybara (Hydrochoerus hydrochaeris: Rodentia, Hydrochoeridae) detectability in anthropogenic environments of southeastern Brazil. Popul. Ecol..

[119] Kugelman J.R., Johnston S.C., Mulembakani P.M., Kisalu N., Lee M.S., Koroleva G., McCarthy S.E., Gestole M.C., Wolfe N.D., Fair J.N. (2014). Genomic variability of monkeypox virus among humans, Democratic Republic of the Congo. Emerg. Infect. Dis..

[120] Nalca A., Zumbrun E.E. (2010). ACAM2000: The new smallpox vaccine for United States Strategic National Stockpile. Drug Des. Dev. Ther..

[121] Olson V.A. (2017). Are we prepared in case of a possible smallpox-like disease emergence?. Viruses.

[122] Quenelle D.C., Prichard M.N., Keith K.A., Hruby D.E., Jordan R., Painter G.R., Robertson A., Kern E.R. (2007). Synergistic efficacy of the combination of ST-246 with CMX001 against orthopoxviruses. Antimicrob. Agents Chemother..

[123] Lederman E.R., Davidson W., Groff H.L., Smith S.K., Warkentien T., Li Y., Wilkins K.A., Karem K.L., Akondy R.S., Ahmed R. (2012). Progressive vaccinia: Case description and laboratory-guided therapy with vaccinia immune globulin, ST-246, and CMX001. J. Infect. Dis..

