# Supplementary material for: Vaccinia Virus Natural Infections in Brazil: The Good, the Bad, and the Ugly
Source: Viruses. 2017 Nov 15;9(11):340. doi: 10.3390/v9110340 (PMC5707547; doi:10.3390/v9110340)
Supplement: Supplementary file 1 [file viruses-09-00340-s001.doc]

**Supplementary material 1**

**Table 1 – A compilation of vaccinia virus detection in Brazil.** The data presented in this table highlight events of VACV natural circulation and infection in different hosts throughout the Brazilian territory.

| **Date of detection** | **Hosts** | **Locality** | **State** | **Methods used** | **BR-VACV Group** | **Reference** | **Study title** |
| --- | --- | --- | --- | --- | --- | --- | --- |
| 1963 | Wild rodents | Belém | Pará | virus isolation, Electron microscopy, PCR | group 2 | Fonseca et al., 1998  [1] | Morphological and molecular characterization of the poxvirus BeAn 58058 |
| 1979 | Sentinel rodents | Cotia | São Paulo | virus isolation, Electron microscopy, PCR | group 2 | Fonseca et al., 2002  [2] | Characterization of a vaccinia-like virus isolated in a Brazilian forest |
| 1993 | Swiss mice | Belo Horizonte | Minas Gerais | animal experimentation, virus isolation, PRNT | no differentiation | Diniz et al., 2001  [3] | An outbreak of mousepox in Swiss mice in a laboratory |
| 1993 | Swiss mice | Belo Horizonte | Minas Gerais | Southern-blot, electron microscopy, PCR, neutralization test, virus isolation | group 2 | Trindade et al., 2004  [4] | Belo Horizonte virus: a vaccinia-like virus lacking the A-type inclusion body gene isolated from infected mice |
| 1995–1996 | Humans | Terra Nova do Norte and Mantena | Mato Grosso and Minas Gerais | PRNT | no differentiation | Figueiredo et al., 2015  [5] | Evaluating anti-Orthopoxvirus antibodies in individuals from Brazilian rural areas prior to the bovine vaccinia era |
| April 1999 | Dairy cattle and humans | Piraí, Aperibé and Cambuci | Rio de Janeiro | virus isolation and electron microscopic, | no differentiation | Schatzmayr et al., 2000  [6] | Detection of Poxvirus in Cattle Associated with Human Cases in the State of Rio de Janeiro: Preliminary Report |
| 1999 | Dairy cattle and humans | Cantagalo, Santo Antonio de Pádua and Miracena | Rio de Janeiro | virus isolation, Western blot, Southern blot, Electron microscopy, PCR | group 1 | Damaso et al., 2000  [7] | An Emergent Poxvirus from Humans and Cattle in Rio de Janeiro State: Cantagalo Virus May Derive from Brazilian Smallpox Vaccine |
| 1999–2007 | Dairy cattle | Cantagalo, Cordeiro, Aperibé, Santo Antonio de Pádua, Cambuci and Miracema | Rio de Janeiro | Virus isolation, electron microscopy, PRNT, PCR | group 1 | Simonetti et al., 2007  [8] | Animal infections by Vaccinia-like viruses in the state of Rio de Janeiro: northwestern region |
| February 2001–September 2002 | Non-human primates | Lajeado and Ipueiras | Tocantis | PRNT, ELISA, PCR | group 1 | Abrahão et al., 2010  [9] | Vaccinia Virus infections in Monkeys, Brazilian Amazon |
| October 2001–July 2003 | Humans | Vale do Paraíba and Vale de São Patrício | São Paulo and Goiás | Direct electron microscopy, Virus isolation, PCR | group 1 | Nagasse-Sugahara et al., 2004  [10] | Human Vaccinia-like virus outbreaks in São Paulo and Goiás states, Brazil: virus detection, isolation and identification |
| October 2001 | Dairy cattle and humans | Guarani | Minas Gerais | Virus isolation, PCR | group 1 and group 2 | Trindade et al., 2006  [11] | Isolation of two Vaccinia Virus strains from a single bovine vaccinia outbreak in rural area from Brazil: implications on the emergence of zoonotic Orthopoxviruses |
| October 2001 | Dairy cattle and humans | Leopoldina, Argirita, Laranjal, Pirapetinga,Volta grande, Cataguases, Astolfo Dutra, Dona Eusébia, Miraí, Santana de Cataguases, Rio Pomba, Guarani, Piau, Piraúba, Ubá, Muriaé, Antônio Prado, Barão de Monte alto, Eugenópolis and Vieiras |  | virus isolation,PRNT, PCR | no differentiation | Lobato et al., 2005  [12] | Surto de varíola bovina causada pelo vírus Vaccinia na região da Zona da Mata Mineira |
| August 2001–May 2005 | Dairy cattle | Alegre, Atílio Vivacgua, Cachoeiro do Itapemirim, Castelo, Itapemirim, Piúma, Presidente Kennedy and Rio Novo do Sul | Espírito Santo | Virus isolation, PCR | no differentiation | Donatele et al., 2007  [13] | Epidemiologia da poxvirose bovina no Estado do Espírito Santo, Brasil |
| 2001–2011 | Dairy cattle, humans and rodents | Aperibé, Cambuci, Cantagalo, Campos dos Goytacazes, Cordeiro, Carmo, Miracema, Itaocara, Sebastião da Paraíba, Barra Mansa, Santo Antônio de Padua, Barra do Piraí, Conservatória, Paraty, Piraí, Resende, Rio Claro, Rio das Flores, Rio de Janeiro, Valença and Paraty | Rio de Janeiro | Virus isolation, electron microscopy, PRNT, PCR | group 1 | Schatzmayr et al., 2011  [14] | Human and animal infections by vaccinia-like viruses in the state of Rio de Janeiro: A novel expanding zoonosis |
| September 2002 | Humans | São Francisco de Itabapoana | Rio de Janeiro | Virus isolation, PRNT, PCR | group 1 | Pereira Oliveira et al., 2014  [15] | Intrafamilial Transmission of Vaccinia virus during a Bovine Vaccinia Outbreak in Brazil: A New Insight in Viral Transmission Chain |
| September 2002–December 2006 | Humans | São Francisco de Itabapoana, Campos dos Goytacazes, Miracema, Cantagalo, São Sebastião do Alto, Cordeiro, Varre-Sai and Natividade | Rio de Janeiro | ELISA, PRNT, PCR | no differentiation | Silva Fernandes et al., 2009  [16] | Natural human infections with Vaccinia virus during bovine vaccinia outbreaks |
| 2003 | Dairy cattle and humans | Araçatuba | São Paulo | Virus Isolation, Electron Microscopy, PCR | group 1 | Trindade et al., 2003  [17] | Araçatuba Virus: A Vaccinialike Virus Associated with Infection in Humans and Cattle |
| March 2003 | Dairy cattle | Passatempo | Minas Gerais | virus isolation, PRNT, electron microscopy,PCR | group 1 | Leite et al., 2005  [18] | Passatempo virus, a Vaccinia virus strain, Brazil |
| 2004 | Humans | Ramal do Granada | Acre | ELISA and PRNT | no differentiation | Mota et al., 2010  [19] | Seroprevalence of orthopoxvirus in an Amazonian rural village, Acre, Brazil |
| 2004–2006 | Capybaras | Cordeiropolis, Cosmorama, Ribeirão Preto, Valparaiso, Andralina | São Paulo | PRNT | no differentiation | Barbosa et al., 2014  [20] | Presence of neutralizing antibodies to Orthopoxvirus in Capybaras (*Hydrochoerus hydrochaeris*) in Brazil |
| June–November 2005 | Dairy cattle and humans | Resplendor | Minas Gerais | PRNT, PCR | group 1 | Abrahão et al., 2010  [21] | Human Vaccinia virus and Pseudocowpox virus co-infection: Clinical description and phylogenetic characterization |
| 2005 | Dairy cattle, humans and rodents | Mariana | Minas Gerais | virus isolation, PRNT, PCR | group 1 | Abrahão et al., 2009  [22] | One more peice in the VACV ecological puzzle: Could peridomestic rodents be the link between wildlife and bovine vaccinia outbreaks in Brazil? |
| 2005 | Dairy cattle | Resplendor | Minas Gerais | virus isolation, PCR, animal experimentation | group 1 | Assis et al., 2012  [23] | Characterization of a New Vaccinia virus Isolate Reveals the C23L Gene as a Putative Genetic Marker for Autochthonous Group 1 Brazilian Vaccinia virus |
| June–November 2005 | Humans | Serro | Minas Gerais | western blot, Virus isolation, PCR | group 1 | Trindade et al., 2009  [24] | Zoonotic Vaccinia Virus: Clinical and Immunological Characteristics in a Naturally Infected Patient |
| 2005–2008 | Milk samples | Bambuí, Paraguaçu and Mariana | Minas Gerais | virus isolation, PCR, Immunofluorescence microscopy | no differentiation | Abrahão et al., 2009  [25] | Bovine Vaccinia Outbreaks: Detection and Isolation of Vaccinia Virus in Milk Samples |
| October–November 2007 | Humans | Itajubá | Minas Gerais | virus isolation, electron microscopy, PRNT and PCR | no differentiation | Silva et al., 2008  [26] | Infecção em humanos por varíola bovina na microrregião de Itajubá, Estado de Minas Gerais: relato de caso |
| February 2008 | Equids | Pelotas | Rio Grande do Sul | Electron microscopy, Histology, PCR | no differentiation | Brum et al., 2010  [27] | An outbreak of orthopoxvirus-associated disease in horses in southern Brazil |
| February 2008 | Equids | Pelotas | Rio Grande do Sul | virus isolation, Animal experimentation, PCR | group 1 and group 2 | Campos et al., 2009  [28] | Assessing the variability of Brazilian Vaccinia virus isolates from a horse exanthematic lesion: coinfection with distinct viruses |
| September 2008 | Dairy cattle and humans | Muricilândia | Tocantis | PCR | group 1 | Medaglia et al., 2009  [29] | Spread of Cantagalo Virus to Northern Brazil |
| Jun 2008–Jun 2010 | Dairy cattle | Ji-Paraná, Jarú, Cocal, Ouro Preto D'Oeste, Ariquemes, Cacaulândia, Teixeirópolis, Nova Brasilândia D’Oeste, Urupá, | Rondônia and Mato Grosso | PCR | group 1 | Quixabeira-Santos et al., 2011  [30] | Animal Movement and Establishment of Vaccinia Virus Cantagalo Strain in Amazon Biome,Brazil |
| February 2009 | Dairy cattle and humans | Açailândia | Maranhão | virus isolation, PCR | group1 | Oliveira et al., 2013  [31] | Group 1 Vaccinia virus Zoonotic Outbreak in Maranhão State, Brazil |
| 2009–2010 | Dairy cattle and humans | Itatinga and Torre de Pedra | São Paulo | ELISA, PRNT, PCR | group 1 | Megid et al., 2012  [32] | Vaccinia Virus zoonotic in São Paulo state, Brazil |
| 2009–2012 | Buffaloes | Cachoeira do Arari, Chaves, Salvaterra, and Soure | Pará | PRNT and PCR | group 1 | Franco-Luiz et al., 2016  [33] | The detection of *Vaccinia virus* confirms the high circulation of *Orthopoxvirus* in buffaloes living in geographical isolation, Marajó Island, Brazilian Amazon |
| June 2010–June 2012 | Dairy cattle and humans | Varjão, Buriti Alegre, São João da Paraúna, Pontalina, Edéia and Mineiros | Goiás | histopathology, PCR, PRNT, ELISA, virus isolation | no differentiation | Sant’Ana et al., 2013  [34] | Outbreaks of vesicular disease caused by Vaccinia virus in dairy cattle from Goiás State, Brazil (2010-2012) |
| 2010 | Dairy cattle | Itatinga | São Paulo | PCR | no differentiation | Antunes et al., 2015  [35] | Intramammary coinfection by vaccinia virus and staphylococcus aureus in a bovine vaccinia outbreak |
| 2010 | Dairy cattle and humans | Doresópolis | Minas Gerais | PRNT, virus isolation, PCR | group 1 | Abrahão et al., 2015  [36] | Outbreak of Severe Zoonotic Vaccinia Virus Infection, Southeastern Brazil |
| July 2010 | Dairy cattle and humans | Bom Jesus do Tocantis | Pará | PRNT, virus isolation, PCR | group 1 | Assis et al., 2013  [37] | Reemergence of Vaccinia Virus during Zoonotic Outbreak, Pará State, Brazil |
| August 2010 | Dairy cattle and humans | Muriaé | Minas Gerais | virus isolation, electron microscopy and PCR | group 1 | Trindade et al., 2007  [38] | Zoonotic Vaccinia Virus Infection in Brazil: Clinical Description and Implications for Health Professionals |
| October 2010 | Buffaloes | Carmo da Mata | Minas Gerais | PRNT | no differentiation | Assis et al., 2012  [39] | Serologic Evidence of Orthopoxvirus Infection in Buffaloes, Brazil |
| August 2011 | Dairy cattle and humans | Carangola | Minas Gerais | virus isolation, PRNT and PCR | group 1 | Assis et al., 2013  [40] | Vaccinia virus in Household Environment during a Bovine Vaccinia Outbreak, Brazil |
| 2011 | Dairy cattle and humans | Serro | Minas Gerais | virus isolation, PCR | Group 2 | Assis et al., 2012  [41] | Group 2 Vaccinia Virus, Brazil |
| 2011 | Dairy cattle, humans, equids, swine, dogs, cats, rodents, marsupials and coatis | Anhembi, Bofete and Torre de Pedra | São Paulo | PRNT and virus neutralization | no differentiation | Peres et al., 2013  [42] | Serological study of vaccinia virus reservoirs in areas with and without official reports of outbreaks in cattle and humans in São Paulo, Brazil |
| 2011 | Dairy cattle and humans |  | Bahia | virus isolation, ELISA, PRNT and PCR | group 1 | Assis et al., 2015  [43] | Horizontal study of vaccinia virus infections in an endemic area: epidemiologic, phylogenetic and economic aspects |
| 2011 | Cattle |  | Minas Gerais | PCR | no differentiation | Rehfeld et al., 2017  [44] | Subclinical bovine vaccinia: An important risk factor in the epidemiology of this zoonosis in cattle |
| May–September 2011 | Wild rodents | Anhembi, Bofete and Torre de Pedra | São Paulo | PCR | no differentiation | Megid et al., 2016  [45] | Vaccinia virus in feces of wild rodents from São Paulo State, Brazil |
| September 2012 | Humans | Serro | Minas Gerais | PRNT and PCR | group 2 | Costa et al., 2015  [46] | Alternative Routes of Zoonotic *Vaccinia Virus* Transmission, Brazil |
| September 2012–December 2014 | Cats |  | Minas Gerais, São Paulo, Rio de Janeiro, Santa Catarina and Ceará | PRNT and PCR | group 1 | Costa et al., 2017  [47] | Detection of Vaccinia Virus in Urban Domestic Cats, Brazil |
| September 2012–March 2013 | Human | Serro | Minas Gerais | PRNT | no differentiation | Costa et al., 2016  [48] | Seroprevalence of Orthopoxvirus in rural Brazil: insights into anti-OPV immunity status and its implications for emergent zoonotic OPV |
| October 2012 | Dogs and opossums | Itatinga | São Paulo | PCR | group 1 | Peres et al., 2016  [49] | Dogs and Opossums Positive for Vaccinia Virus during Outbreak Affecting Cattle and Humans São Paulo State, Brazil |
| December 2012–May 2013 | Capybaras | Belo Horizonte, Serra do Cipó and Pantanal | Minas Gerais and Mato Grosso do Sul | PCR | group 1 | Dutra et al., 2017  [50] | Molecular evidence of Orthopoxvirus DNA in capybara Molecular evidence of Orthopoxvirus DNA in capybara (*Hydrochoerus hydrochaeri*s) stool samples |
| April 2011–September 2013 | Wild rodents and marsupials | Sabará, Serro and Rio Pomba | Minas Gerais | ELISA, PRNT, PCR | group 1 and group 2 | Miranda et al., 2017  [51] | Serologic and Molecular Evidence of Vaccinia Virus Circulation among Small Mammals Serologic and Molecular Evidence of Vaccinia Virus Circulation among Small Mammals from Different Biomes, Brazil |
| August 2014 | Equids | Itiuba, Santa Luz | Bahia | PRNT, PCR | group 1 | Abrahão et al., 2017  [52] | Detection of *Vaccinia virus* during an outbreak of exanthemous oral lesions in Brazilian equids |

**References:**

1. Fonseca F.G., Lanna M.C., Campos M.A., Kitajima E.W., Peres J.N., Golgher R.R., et al. *Morphological and molecular characterization of the poxvirus BeAn 58058.* Arch. Virol. 1998;143:1171-86.

2. Da Fonseca F.G., Trindade G.S., Silva R.L., Bonjardim C.A., Ferreira P.C., Kroon E.G. *Characterization of a vaccinia-like virus isolated in a Brazilian forest.* J. Gen. Virol. 2002;83:223–228.

3. Diniz S., Trindade G.S., Fonseca F.G., Kroon E.G. *An outbreak of mousepox in swiss mice in a laboratory animal facility – Case report.* Arq. Bras. Med. Vet. Zootec. 2001;53:152-6.

4. Trindade G.S., da Fonseca F.G., Marques J.T., Diniz S., Leite J.A., de Bodt S., et al. *Belo Horizonte virus: a vaccinia-like virus lacking A-type inclusion body gene isolated from infected mice.* J. Gen. Virol. 2004;85:2015-21.

5. Figueiredo P.O., Silva-Fernandes A.T., Mota B.E., Costa G.B., Borges I.A., Ferreira P.C., et al. *Evaluating anti-Orthopoxvirus antibodies in individuals from Brazilian rural areas prior to the bovine vaccinia era.* Mem. Inst. Oswaldo Cruz. 2015;110:804-8.

6. Schatzmayr H.G., Lemos E.R., Mazur C., Schubach A., Majerowicz S., Rozental T., et al. *Detection of Poxvirus in Cattle Associated with Human Cases in the State of Rio de Janeiro: Preliminary Report.* Mem. Inst. Oswaldo Cruz. 2000;95:625-7.

7. Damaso C.R., Esposito J.J., Condit R.C., Moussatche N. *An emergent poxvirus from humans and cattle in Rio de Janeiro State: Cantagalo virus may derive from Brazilian smallpox vaccine.* Virology 2000;277:439–449.

8. Schatzmayr H.G., Simonetti B.R., Abreu D.C., Simonetti J.P., Simonetti S.R., Costa R.V.C., et al. *Animal infections by Vaccinia-like viruses in the state of Rio de Janeiro: northwestern region.* Pesq. Vet. Bras. 2009;29:509-14.

9. Abrahão J.S., Silva-Fernandes A.T., Lima L.S., Campos R.K., Guedes M.I., Cota M.M., et al. *Vaccinia Virus infections in Monkeys, Brazilian Amazon.* Emerg. Infect. Dis. 2010;16:976-9.

10. Nagasse-Sugahara T.K., Kisielius J.J., Ueda-Ito M., Curti S.P., Figueiredo C.A., Cruz A.S., et al. *Human Vaccinia-like virus outbreaks in São Paulo and Goiás states, Brazil: virus detection, isolation and identification.* Rev. Inst. Med. Trop. São Paulo. 2004;46:315-22.

11. Trindade G.S., Lobato Z.I., Drumond B.P., Leite J.A., Trigueiro R.C., Guedes M.I., et al. *Short report: Isolation of two vaccinia virus strains from a single bovine vaccinia outbreak in rural area from Brazil: Implications on the emergence of zoonotic orthopoxviruses.* Am. J. Trop. Med. Hyg. 2006;75:486–490.

12. Lobato Z.I.P., Trindade G.S., Frois M.C.M., Ribeiro E.B.T., Dias G.R.C., Teixeira B.M., et al. *Outbreak of exantemal disease caused by Vaccinia virus in human and cattle in Zona da Mata region, Minas Gerais.* Arq. Bras. Med. Vet. Zootec. 2005;57:423-9.

13. Donatele D.M., Travassos C.E.P.F., Leite J.A., Kroon E.G. *Epidemiologia da poxvirose bovina no Estado do Espírito Santo, Brasil.* Braz. J. Vet. Res. Anim. Sci. 2007;44:275-82.

14. Schatzmayr H.G., Costa R.V., [Gonçalves M.C](https://www.ncbi.nlm.nih.gov/pubmed/?term=Gon%C3%A7alves%20MC%5BAuthor%5D&cauthor=true&cauthor_uid=22185829)., M.C., D’Andrea P.S., Barth O.M. *Human and animal infections by vaccinia-like viruses in the state of Rio de Janeiro: A novel expanding zoonosis.* Vaccine. 2011;Suppl 4:D65-9.

15. [Pereira Oliveira G](https://www.ncbi.nlm.nih.gov/pubmed/?term=Pereira%20Oliveira%20G%5BAuthor%5D&cauthor=true&cauthor_uid=24615135)., [Tavares Silva Fernandes A](https://www.ncbi.nlm.nih.gov/pubmed/?term=Tavares%20Silva%20Fernandes%20A%5BAuthor%5D&cauthor=true&cauthor_uid=24615135)., [Lopes de Assis F](https://www.ncbi.nlm.nih.gov/pubmed/?term=Lopes%20de%20Assis%20F%5BAuthor%5D&cauthor=true&cauthor_uid=24615135)., [Augusto Alves P](https://www.ncbi.nlm.nih.gov/pubmed/?term=Augusto%20Alves%20P%5BAuthor%5D&cauthor=true&cauthor_uid=24615135)., [Moreira Franco Luiz AP](https://www.ncbi.nlm.nih.gov/pubmed/?term=Moreira%20Franco%20Luiz%20AP%5BAuthor%5D&cauthor=true&cauthor_uid=24615135)., [Barcelos Figueiredo L](https://www.ncbi.nlm.nih.gov/pubmed/?term=Barcelos%20Figueiredo%20L%5BAuthor%5D&cauthor=true&cauthor_uid=24615135)., et al. *Intrafamilial Transmission of* Vaccinia virus *during a Bovine Vaccinia Outbreak in Brazil: A New Insight in Viral Transmission Chain.* Am. J. Trop. Med. Hyg. 2014;90:1021-3.

16. Silva-Fernandes A.T., Travassos C.E., Ferreira J.M., Abrahão J.S., Rocha E.S., Viana-Ferreira F., et al. *Natural human infections with Vaccinia virus during bovine vaccinia outbreaks.* J. Clin. Virol. 2009;44:308-13.

17. De Souza Trindade G., da Fonseca F.G., Marques J.T., Nogueira M.L., Mendes L.C.N., Borges A.S., Peiró J.R., Pituco E.M., Bonjardim C.A., Ferreira P.C.P., et al. *Araçatuba virus: A vaccinia-like virus associated with infection in humans and cattle.* Emerg. Infect. Dis. 2003;9:155–160.

18. Leite J.A., Drumond B.P., Trindade G.S., Lobato Z.I., da Fonseca F.G., Madureira M.C., Guedes M.I., Ferreira J.M., Bonjardim C.A., Ferreira P.C., et al. *Passatempo virus, a vaccinia virus strain, Brazil.* Emerg. Infect. Dis. 2005;11:1935–1938.

19. [Mota B.E](https://www.ncbi.nlm.nih.gov/pubmed/?term=Mota%20BE%5BAuthor%5D&cauthor=true&cauthor_uid=20428903)., [Trindade G.S](https://www.ncbi.nlm.nih.gov/pubmed/?term=Trindade%20GS%5BAuthor%5D&cauthor=true&cauthor_uid=20428903)., [Diniz T.C](https://www.ncbi.nlm.nih.gov/pubmed/?term=Diniz%20TC%5BAuthor%5D&cauthor=true&cauthor_uid=20428903)., [da Silva-Nunes M](https://www.ncbi.nlm.nih.gov/pubmed/?term=da%20Silva-Nunes%20M%5BAuthor%5D&cauthor=true&cauthor_uid=20428903)., [Braga E.M](https://www.ncbi.nlm.nih.gov/pubmed/?term=Braga%20EM%5BAuthor%5D&cauthor=true&cauthor_uid=20428903)., [Urbano-Ferreira M](https://www.ncbi.nlm.nih.gov/pubmed/?term=Urbano-Ferreira%20M%5BAuthor%5D&cauthor=true&cauthor_uid=20428903)., et al. *Seroprevalence of orthopoxvirus in an Amazonian rural village, Acre, Brazil.* Arch. Virol. 2010;155:1139-44.

20. Barbosa A.V., Medaglia M.L., Soares H.S., Quixabeira-Santos J.C., Gennari S.M., Damaso C.R. *Presence of neutralizing antibodies to Orthopoxvirus in Capybaras (Hydrochoerus hydrochaeris) in Brazil.* J. Infect. Dev. Ctries. 2014;8:1646-9.

21. [Abrahão J.S](https://www.ncbi.nlm.nih.gov/pubmed/?term=Abrah%C3%A3o%20JS%5BAuthor%5D&cauthor=true&cauthor_uid=20207192)., [Silva-Fernandes A.T](https://www.ncbi.nlm.nih.gov/pubmed/?term=Silva-Fernandes%20AT%5BAuthor%5D&cauthor=true&cauthor_uid=20207192)., [Assis F.L](https://www.ncbi.nlm.nih.gov/pubmed/?term=Assis%20FL%5BAuthor%5D&cauthor=true&cauthor_uid=20207192)., [Guedes M.I](https://www.ncbi.nlm.nih.gov/pubmed/?term=Guedes%20MI%5BAuthor%5D&cauthor=true&cauthor_uid=20207192)., [Drumond B.P](https://www.ncbi.nlm.nih.gov/pubmed/?term=Drumond%20BP%5BAuthor%5D&cauthor=true&cauthor_uid=20207192)., [Leite J.A](https://www.ncbi.nlm.nih.gov/pubmed/?term=Leite%20JA%5BAuthor%5D&cauthor=true&cauthor_uid=20207192)., et al. *Human Vaccinia virus and Pseudocowpox virus co-infection: Clinical description and phylogenetic characterization.* J. Clin. Virol. 2010;48:69-72.

22. Abrahão J.S., Guedes M.I., Trindade G.S., Fonseca F.G., Campos R.K., Mota B.F., Lobato Z.I., Silva-Fernandes A.T., Rodrigues G.O., Lima L.S., et al. *One More Piece in the VACV Ecological Puzzle: Could Peridomestic Rodents Be the Link between Wildlife and Bovine Vaccinia Outbreaks in Brazil?* PLoS ONE. 2009;4:e7428.

23. [Assis F.L](https://www.ncbi.nlm.nih.gov/pubmed/?term=Assis%20FL%5BAuthor%5D&cauthor=true&cauthor_uid=23189200)., [Almeida G.M](https://www.ncbi.nlm.nih.gov/pubmed/?term=Almeida%20GM%5BAuthor%5D&cauthor=true&cauthor_uid=23189200)., [Oliveira D.B](https://www.ncbi.nlm.nih.gov/pubmed/?term=Oliveira%20DB%5BAuthor%5D&cauthor=true&cauthor_uid=23189200)., [Franco-Luiz A.P](https://www.ncbi.nlm.nih.gov/pubmed/?term=Franco-Luiz%20AP%5BAuthor%5D&cauthor=true&cauthor_uid=23189200)., [Campos R.K](https://www.ncbi.nlm.nih.gov/pubmed/?term=Campos%20RK%5BAuthor%5D&cauthor=true&cauthor_uid=23189200)., [Guedes M.I](https://www.ncbi.nlm.nih.gov/pubmed/?term=Guedes%20MI%5BAuthor%5D&cauthor=true&cauthor_uid=23189200)., et al. *Characterization of a New Vaccinia virus Isolate Reveals the C23L Gene as a Putative Genetic Marker for Autochthonous Group 1 Brazilian Vaccinia virus.* PLoS One. 2012;7:e50413.

24. Trindade G.S., Guedes M.I., Drumond B.P., Mota B.E., Abrahão J.S., Lobato Z.I., Gomes J.A., Corrêa-Oliveira R., Nogueira M.L., Kroon E.G., et al. *Zoonotic vaccinia virus: Clinical and immunological characteristics in a naturally infected patient.* Clin. Infect. Dis. 2009;3:37–40.

25. Abrahão J.S., Oliveira T.M., Campos R.K., Madureira M.C., Kroon E.G., Lobato Z.I. *Bovine Vaccinia Outbreaks: Detection and Isolation of Vaccinia Virus in Milk Samples.* Foodborne Pathog. Dis. 2009;6:1141-6.

26. da Silva A.C., Reis B.B., Ricci Junior J.E.R., Fernandes F.S., Corrêa F.J., Schatzmayr H.G. *Infecção em humanos por varíola bovina na microrregião de Itajubá, Estado de Minas Gerais: relato de caso.* Rev. Soc. Bras. Med. Trop. 2008;41:507-11.

27. [Brum M.C](https://www.ncbi.nlm.nih.gov/pubmed/?term=Brum%20MC%5BAuthor%5D&cauthor=true&cauthor_uid=20093706)., [Anjos B.L](https://www.ncbi.nlm.nih.gov/pubmed/?term=Anjos%20BL%5BAuthor%5D&cauthor=true&cauthor_uid=20093706)., [Nogueira C.E](https://www.ncbi.nlm.nih.gov/pubmed/?term=Nogueira%20CE%5BAuthor%5D&cauthor=true&cauthor_uid=20093706)., [Amaral L.A](https://www.ncbi.nlm.nih.gov/pubmed/?term=Amaral%20LA%5BAuthor%5D&cauthor=true&cauthor_uid=20093706)., [Weiblen R](https://www.ncbi.nlm.nih.gov/pubmed/?term=Weiblen%20R%5BAuthor%5D&cauthor=true&cauthor_uid=20093706)., [Flores E.F](https://www.ncbi.nlm.nih.gov/pubmed/?term=Flores%20EF%5BAuthor%5D&cauthor=true&cauthor_uid=20093706). *An outbreak of orthopoxvirus-associated disease in horses in southern Brazil.* J. Vet. Diagn. Invest. 2010;22:143-7.

28. [Campos R.K](https://www.ncbi.nlm.nih.gov/pubmed/?term=Campos%20RK%5BAuthor%5D&cauthor=true&cauthor_uid=21080203)., [Brum M.C](https://www.ncbi.nlm.nih.gov/pubmed/?term=Brum%20MC%5BAuthor%5D&cauthor=true&cauthor_uid=21080203)., [Nogueira C.E](https://www.ncbi.nlm.nih.gov/pubmed/?term=Nogueira%20CE%5BAuthor%5D&cauthor=true&cauthor_uid=21080203)., [Drumond B.P](https://www.ncbi.nlm.nih.gov/pubmed/?term=Drumond%20BP%5BAuthor%5D&cauthor=true&cauthor_uid=21080203)., [Alves P.A](https://www.ncbi.nlm.nih.gov/pubmed/?term=Alves%20PA%5BAuthor%5D&cauthor=true&cauthor_uid=21080203)., [Siqueira-Lima L](https://www.ncbi.nlm.nih.gov/pubmed/?term=Siqueira-Lima%20L%5BAuthor%5D&cauthor=true&cauthor_uid=21080203)., et al. *Assessing the variability of Brazilian Vaccinia virus isolates from a horse exanthematic lesion: coinfection with distinct viruses.* Arch. Virol. 2011;156:275-83.

29. [Medaglia M.L](https://www.ncbi.nlm.nih.gov/pubmed/?term=Medaglia%20ML%5BAuthor%5D&cauthor=true&cauthor_uid=19624947)., [Pessoa L.C](https://www.ncbi.nlm.nih.gov/pubmed/?term=Pessoa%20LC%5BAuthor%5D&cauthor=true&cauthor_uid=19624947)., [Sales E.R](https://www.ncbi.nlm.nih.gov/pubmed/?term=Sales%20ER%5BAuthor%5D&cauthor=true&cauthor_uid=19624947)., [Freitas T.R](https://www.ncbi.nlm.nih.gov/pubmed/?term=Freitas%20TR%5BAuthor%5D&cauthor=true&cauthor_uid=19624947)., [Damaso C.R](https://www.ncbi.nlm.nih.gov/pubmed/?term=Damaso%20CR%5BAuthor%5D&cauthor=true&cauthor_uid=19624947). *Spread of Cantagalo Virus to Northern Brazil.* Emerg. Infect. Dis. 2009, 15, 1142-3.

30. Quixabeira-Santos J.C., Medaglia M.L., Pescador C.A., Damaso C.R. *Animal movement and establishment of vaccinia virus Cantagalo strain in Amazon biome, Brazil.* Emerg. Infect. Dis. 2011;4:726–729.

31. [Oliveira D.B](https://www.ncbi.nlm.nih.gov/pubmed/?term=Oliveira%20DB%5BAuthor%5D&cauthor=true&cauthor_uid=24166043)., [Assis F.L](https://www.ncbi.nlm.nih.gov/pubmed/?term=Assis%20FL%5BAuthor%5D&cauthor=true&cauthor_uid=24166043)., [Ferreira P.C](https://www.ncbi.nlm.nih.gov/pubmed/?term=Ferreira%20PC%5BAuthor%5D&cauthor=true&cauthor_uid=24166043)., [Bonjardim C.A](https://www.ncbi.nlm.nih.gov/pubmed/?term=Bonjardim%20CA%5BAuthor%5D&cauthor=true&cauthor_uid=24166043)., [de Souza Trindade G](https://www.ncbi.nlm.nih.gov/pubmed/?term=de%20Souza%20Trindade%20G%5BAuthor%5D&cauthor=true&cauthor_uid=24166043)., [Kroon E.G](https://www.ncbi.nlm.nih.gov/pubmed/?term=Kroon%20EG%5BAuthor%5D&cauthor=true&cauthor_uid=24166043)., [Abrahão J.S](https://www.ncbi.nlm.nih.gov/pubmed/?term=Abrah%C3%A3o%20JS%5BAuthor%5D&cauthor=true&cauthor_uid=24166043). *Group 1 Vaccinia virus Zoonotic Outbreak in Maranhão State, Brazil.* Am. J. Trop. Med. Hyg. 2013;89:1142-5.

32. [Megid J](https://www.ncbi.nlm.nih.gov/pubmed/?term=Megid%20J%5BAuthor%5D&cauthor=true&cauthor_uid=22260819)., [Borges I.A](https://www.ncbi.nlm.nih.gov/pubmed/?term=Borges%20IA%5BAuthor%5D&cauthor=true&cauthor_uid=22260819)., [Abrahão J.S](https://www.ncbi.nlm.nih.gov/pubmed/?term=Abrah%C3%A3o%20JS%5BAuthor%5D&cauthor=true&cauthor_uid=22260819)., [Trindade G.S](https://www.ncbi.nlm.nih.gov/pubmed/?term=Trindade%20GS%5BAuthor%5D&cauthor=true&cauthor_uid=22260819)., [Appolinário C.M](https://www.ncbi.nlm.nih.gov/pubmed/?term=Appolin%C3%A1rio%20CM%5BAuthor%5D&cauthor=true&cauthor_uid=22260819)., [Ribeiro M.G](https://www.ncbi.nlm.nih.gov/pubmed/?term=Ribeiro%20MG%5BAuthor%5D&cauthor=true&cauthor_uid=22260819)., et al. *Vaccinia Virus zoonotic in São Paulo state, Brazil.* Emerg. Infect. Dise. 2012;18:189-91.

33. [Franco-Luiz A.P](https://www.ncbi.nlm.nih.gov/pubmed/?term=Franco-Luiz%20AP%5BAuthor%5D&cauthor=true&cauthor_uid=27260805)., [Fagundes Pereira A](https://www.ncbi.nlm.nih.gov/pubmed/?term=Fagundes%20Pereira%20A%5BAuthor%5D&cauthor=true&cauthor_uid=27260805)., [de Oliveira C.H](https://www.ncbi.nlm.nih.gov/pubmed/?term=de%20Oliveira%20CH%5BAuthor%5D&cauthor=true&cauthor_uid=27260805)., [Barbosa J.D](https://www.ncbi.nlm.nih.gov/pubmed/?term=Barbosa%20JD%5BAuthor%5D&cauthor=true&cauthor_uid=27260805)., [Oliveira D.B](https://www.ncbi.nlm.nih.gov/pubmed/?term=Oliveira%20DB%5BAuthor%5D&cauthor=true&cauthor_uid=27260805)., [Bonjardim C.A](https://www.ncbi.nlm.nih.gov/pubmed/?term=Bonjardim%20CA%5BAuthor%5D&cauthor=true&cauthor_uid=27260805)., et al. *The detection of Vaccinia virus confirms the high circulation of Orthopoxvirus in buffaloes living in geographical isolation, Marajó Island, Brazilian Amazon.* Comp. Immunol. Microbiol. Infect. Dis. 2016;46:16-9.

34. Sant’Anna F.J.F. Leal A.A., Rabelo R.E., Vulcani V.A.S., Ferreira Junior J.A., Cargnelutti J.F., Flores E.F. *Outbreaks of vesicular disease caused by Vaccinia virus in dairy cattle from Goiás State, Brazil (2010-2012).* Pesq. Vet. Bras. 2013;33:860-6.

35. Antunes J.M.A.P., Ribeiro M.G., Megid J. *Intramammary coinfection by vaccinia virus and staphylococcus aureus in a bovine vaccinia outbreak.* JMM Case Reports. 2015;2:1-4.

36. Abrahão J.S., Campos R.K., Trindade G.S., Guimarães da Fonseca F., Ferreira P.C., Kroon E.G. *Outbreak of Severe Zoonotic Vaccinia Virus Infection, Southeastern Brazil.* Emerg. Infect. Dis. 2015;21:695-8.

37. [de Assis F.L](https://www.ncbi.nlm.nih.gov/pubmed/?term=de%20Assis%20FL%5BAuthor%5D&cauthor=true&cauthor_uid=24274374)., [Vinhote W.M](https://www.ncbi.nlm.nih.gov/pubmed/?term=Vinhote%20WM%5BAuthor%5D&cauthor=true&cauthor_uid=24274374)., [Barbosa J.D](https://www.ncbi.nlm.nih.gov/pubmed/?term=Barbosa%20JD%5BAuthor%5D&cauthor=true&cauthor_uid=24274374)., [de Oliveira C.H](https://www.ncbi.nlm.nih.gov/pubmed/?term=de%20Oliveira%20CH%5BAuthor%5D&cauthor=true&cauthor_uid=24274374)., [de Oliveira C.M](https://www.ncbi.nlm.nih.gov/pubmed/?term=de%20Oliveira%20CM%5BAuthor%5D&cauthor=true&cauthor_uid=24274374)., [Campos K.F](https://www.ncbi.nlm.nih.gov/pubmed/?term=Campos%20KF%5BAuthor%5D&cauthor=true&cauthor_uid=24274374)., et al. *Reemergence of Vaccinia Virus during Zoonotic Outbreak, Pará State, Brazil.* Emerg. Infect. Dis. 2013;19:2017-20.

38. De Souza Trindade G., Drumond B.P., Guedes M.I., Leite J.A., Mota B.E. *Zoonotic vaccinia virus infection in Brazil: Clinical description and implications for health professionals.* J. Clin. Microbiol. 2007;4:1370–1372.

39. [de Assis FL](https://www.ncbi.nlm.nih.gov/pubmed/?term=de%20Assis%20FL%5BAuthor%5D&cauthor=true&cauthor_uid=22469217), [Pereira G](https://www.ncbi.nlm.nih.gov/pubmed/?term=Pereira%20G%5BAuthor%5D&cauthor=true&cauthor_uid=22469217), [Oliveira C](https://www.ncbi.nlm.nih.gov/pubmed/?term=Oliveira%20C%5BAuthor%5D&cauthor=true&cauthor_uid=22469217), [Rodrigues GO](https://www.ncbi.nlm.nih.gov/pubmed/?term=Rodrigues%20GO%5BAuthor%5D&cauthor=true&cauthor_uid=22469217), [Cotta MM](https://www.ncbi.nlm.nih.gov/pubmed/?term=Cotta%20MM%5BAuthor%5D&cauthor=true&cauthor_uid=22469217), [Silva-Fernandes AT](https://www.ncbi.nlm.nih.gov/pubmed/?term=Silva-Fernandes%20AT%5BAuthor%5D&cauthor=true&cauthor_uid=22469217), [Ferreira PC](https://www.ncbi.nlm.nih.gov/pubmed/?term=Ferreira%20PC%5BAuthor%5D&cauthor=true&cauthor_uid=22469217), [Bonjardim CA](https://www.ncbi.nlm.nih.gov/pubmed/?term=Bonjardim%20CA%5BAuthor%5D&cauthor=true&cauthor_uid=22469217), [Trindade Gde S](https://www.ncbi.nlm.nih.gov/pubmed/?term=Trindade%20Gde%20S%5BAuthor%5D&cauthor=true&cauthor_uid=22469217), [Kroon EG](https://www.ncbi.nlm.nih.gov/pubmed/?term=Kroon%20EG%5BAuthor%5D&cauthor=true&cauthor_uid=22469217), [Abrahão JS](https://www.ncbi.nlm.nih.gov/pubmed/?term=Abrah%C3%A3o%20JS%5BAuthor%5D&cauthor=true&cauthor_uid=22469217). *Serologic Evidence of Orthopoxvirus Infection in Buffaloes, Brazil.* Emerg. Infect. Dis. 2012;18:698-700.

40. [Assis F.L](https://www.ncbi.nlm.nih.gov/pubmed/?term=Assis%20FL%5BAuthor%5D&cauthor=true&cauthor_uid=24274787)., [Borges I.A](https://www.ncbi.nlm.nih.gov/pubmed/?term=Borges%20IA%5BAuthor%5D&cauthor=true&cauthor_uid=24274787)., [Mesquita V.S](https://www.ncbi.nlm.nih.gov/pubmed/?term=Mesquita%20VS%5BAuthor%5D&cauthor=true&cauthor_uid=24274787)., [Ferreira P.C](https://www.ncbi.nlm.nih.gov/pubmed/?term=Ferreira%20PC%5BAuthor%5D&cauthor=true&cauthor_uid=24274787)., [Trindade G.S](https://www.ncbi.nlm.nih.gov/pubmed/?term=Trindade%20GS%5BAuthor%5D&cauthor=true&cauthor_uid=24274787)., [Kroon E.G](https://www.ncbi.nlm.nih.gov/pubmed/?term=Kroon%20EG%5BAuthor%5D&cauthor=true&cauthor_uid=24274787)., [Abrahão J.S](https://www.ncbi.nlm.nih.gov/pubmed/?term=Abrah%C3%A3o%20JS%5BAuthor%5D&cauthor=true&cauthor_uid=24274787). *Vaccinia virus in Household Environment during a Bovine Vaccinia Outbreak, Brazil.* Emerg. Infect. Dis. 2013;19:2045-7.

41. Assis F.L., Borges I.A., Ferreira P.C., Bonjardim C.A., Trindade G.S., Lobato Z.I., et al. *Group 2 Vaccinia Virus, Brazil.* Emerg. Infect. Dis. 2012;18:2035-8.

42. [Peres M.G](https://www.ncbi.nlm.nih.gov/pubmed/?term=Peres%20MG%5BAuthor%5D&cauthor=true&cauthor_uid=23760628)., [Bacchiega T.S](https://www.ncbi.nlm.nih.gov/pubmed/?term=Bacchiega%20TS%5BAuthor%5D&cauthor=true&cauthor_uid=23760628)., [Appolinário C.M](https://www.ncbi.nlm.nih.gov/pubmed/?term=Appolin%C3%A1rio%20CM%5BAuthor%5D&cauthor=true&cauthor_uid=23760628)., [Vicente A.F](https://www.ncbi.nlm.nih.gov/pubmed/?term=Vicente%20AF%5BAuthor%5D&cauthor=true&cauthor_uid=23760628)., [Allendorf S.D](https://www.ncbi.nlm.nih.gov/pubmed/?term=Allendorf%20SD%5BAuthor%5D&cauthor=true&cauthor_uid=23760628)., [Antunes J.M](https://www.ncbi.nlm.nih.gov/pubmed/?term=Antunes%20JM%5BAuthor%5D&cauthor=true&cauthor_uid=23760628)., et al. *Serological study of vaccinia virus reservoirs in areas with and without official reports of outbreaks in cattle and humans in São Paulo, Brazil.* Arch. Virol. 2013;158:2433-41.

43. [Assis F.L](https://www.ncbi.nlm.nih.gov/pubmed/?term=Assis%20FL%5BAuthor%5D&cauthor=true&cauthor_uid=26239343)., [Franco-Luiz A.P](https://www.ncbi.nlm.nih.gov/pubmed/?term=Franco-Luiz%20AP%5BAuthor%5D&cauthor=true&cauthor_uid=26239343)., [Paim L.M](https://www.ncbi.nlm.nih.gov/pubmed/?term=Paim%20LM%5BAuthor%5D&cauthor=true&cauthor_uid=26239343)., [Oliveira G.P](https://www.ncbi.nlm.nih.gov/pubmed/?term=Oliveira%20GP%5BAuthor%5D&cauthor=true&cauthor_uid=26239343)., [Pereira A.F](https://www.ncbi.nlm.nih.gov/pubmed/?term=Pereira%20AF%5BAuthor%5D&cauthor=true&cauthor_uid=26239343)., [de Almeida G.M](https://www.ncbi.nlm.nih.gov/pubmed/?term=de%20Almeida%20GM%5BAuthor%5D&cauthor=true&cauthor_uid=26239343)., [Figueiredo L.B](https://www.ncbi.nlm.nih.gov/pubmed/?term=Figueiredo%20LB%5BAuthor%5D&cauthor=true&cauthor_uid=26239343)., et al. *Horizontal study of vaccinia virus infections in an endemic area: epidemiologic, phylogenetic and economic aspects.* Arch. Virol. 2015;160:2703-8.

44. [Rehfeld I.S](https://www.ncbi.nlm.nih.gov/pubmed/?term=Rehfeld%20IS%5BAuthor%5D&cauthor=true&cauthor_uid=28502903)., [Matos A.C.D](https://www.ncbi.nlm.nih.gov/pubmed/?term=Matos%20ACD%5BAuthor%5D&cauthor=true&cauthor_uid=28502903)., [Guedes M.I.M.C](https://www.ncbi.nlm.nih.gov/pubmed/?term=Guedes%20MIMC%5BAuthor%5D&cauthor=true&cauthor_uid=28502903)., [Costa A.G](https://www.ncbi.nlm.nih.gov/pubmed/?term=Costa%20AG%5BAuthor%5D&cauthor=true&cauthor_uid=28502903)., [Fraiha A.L.S](https://www.ncbi.nlm.nih.gov/pubmed/?term=Fraiha%20ALS%5BAuthor%5D&cauthor=true&cauthor_uid=28502903)., [Lobato Z.I.P](https://www.ncbi.nlm.nih.gov/pubmed/?term=Lobato%20ZIP%5BAuthor%5D&cauthor=true&cauthor_uid=28502903). *Subclinical bovine vaccinia: An important risk factor in the epidemiology of this zoonosis in cattle.* Res. Vet. Sci. 2017;114:233-5.

45. Megid J., Peres M.G., Bachiega T., Appolinario C.M., Vicente A.F., Ribeiro B.L.D., et al. *Vaccinia virus in feces of wild rodents from São Paulo State, Brazil.* Int. J. Infect. Dis. 2016;53S:128.

46. [Costa G.B](https://www.ncbi.nlm.nih.gov/pubmed/?term=Costa%20GB%5BAuthor%5D&cauthor=true&cauthor_uid=26583465)., [Borges I.A](https://www.ncbi.nlm.nih.gov/pubmed/?term=Borges%20IA%5BAuthor%5D&cauthor=true&cauthor_uid=26583465)., [Alves P.A](https://www.ncbi.nlm.nih.gov/pubmed/?term=Alves%20PA%5BAuthor%5D&cauthor=true&cauthor_uid=26583465)., [Miranda J.B](https://www.ncbi.nlm.nih.gov/pubmed/?term=Miranda%20JB%5BAuthor%5D&cauthor=true&cauthor_uid=26583465)., [Luiz A.P](https://www.ncbi.nlm.nih.gov/pubmed/?term=Luiz%20AP%5BAuthor%5D&cauthor=true&cauthor_uid=26583465)., [Ferreira P.C](https://www.ncbi.nlm.nih.gov/pubmed/?term=Ferreira%20PC%5BAuthor%5D&cauthor=true&cauthor_uid=26583465)., et al. *Alternative Routes of Zoonotic Vaccinia Virus Transmission, Brazil.* Emerg. Infect. Dis. 2015;21:2244-6.

47. [Costa G.B](https://www.ncbi.nlm.nih.gov/pubmed/?term=Costa%20GB%5BAuthor%5D&cauthor=true&cauthor_uid=28098542)., [Miranda J.B](https://www.ncbi.nlm.nih.gov/pubmed/?term=Miranda%20JB%5BAuthor%5D&cauthor=true&cauthor_uid=28098542)., [Almeida G.G](https://www.ncbi.nlm.nih.gov/pubmed/?term=Almeida%20GG%5BAuthor%5D&cauthor=true&cauthor_uid=28098542)., [Silva de Oliveira J](https://www.ncbi.nlm.nih.gov/pubmed/?term=Silva%20de%20Oliveira%20J%5BAuthor%5D&cauthor=true&cauthor_uid=28098542)., [Pinheiro M.S](https://www.ncbi.nlm.nih.gov/pubmed/?term=Pinheiro%20MS%5BAuthor%5D&cauthor=true&cauthor_uid=28098542)., [Gonçalves S.A](https://www.ncbi.nlm.nih.gov/pubmed/?term=Gon%C3%A7alves%20SA%5BAuthor%5D&cauthor=true&cauthor_uid=28098542)., et al. *Detection of Vaccinia Virus in Urban Domestic Cats, Brazil.* Emerg. Infect. Dis. 2017;23:360-2.

48. [Costa G.B](https://www.ncbi.nlm.nih.gov/pubmed/?term=Costa%20GB%5BAuthor%5D&cauthor=true&cauthor_uid=27377374)., [Augusto L.T](https://www.ncbi.nlm.nih.gov/pubmed/?term=Augusto%20LT%5BAuthor%5D&cauthor=true&cauthor_uid=27377374)., [Leite J.A](https://www.ncbi.nlm.nih.gov/pubmed/?term=Leite%20JA%5BAuthor%5D&cauthor=true&cauthor_uid=27377374)., [Ferreira P.C](https://www.ncbi.nlm.nih.gov/pubmed/?term=Ferreira%20PC%5BAuthor%5D&cauthor=true&cauthor_uid=27377374)., [Bonjardim C.A](https://www.ncbi.nlm.nih.gov/pubmed/?term=Bonjardim%20CA%5BAuthor%5D&cauthor=true&cauthor_uid=27377374)., [Abrahão J.S](https://www.ncbi.nlm.nih.gov/pubmed/?term=Abrah%C3%A3o%20JS%5BAuthor%5D&cauthor=true&cauthor_uid=27377374)., et al. *Seroprevalence of Orthopoxvirus in rural Brazil: insights into anti-OPV immunity status and its implications for emergent zoonotic OPV.* Virol. J. 2016;13:121.

49. [Peres M.G](https://www.ncbi.nlm.nih.gov/pubmed/?term=Peres%20MG%5BAuthor%5D&cauthor=true&cauthor_uid=26812352)., [Barros C.B](https://www.ncbi.nlm.nih.gov/pubmed/?term=Barros%20CB%5BAuthor%5D&cauthor=true&cauthor_uid=26812352)., [Appolinário C.M](https://www.ncbi.nlm.nih.gov/pubmed/?term=Appolin%C3%A1rio%20CM%5BAuthor%5D&cauthor=true&cauthor_uid=26812352)., [Antunes J.M](https://www.ncbi.nlm.nih.gov/pubmed/?term=Antunes%20JM%5BAuthor%5D&cauthor=true&cauthor_uid=26812352)., [Mioni M.S](https://www.ncbi.nlm.nih.gov/pubmed/?term=Mioni%20MS%5BAuthor%5D&cauthor=true&cauthor_uid=26812352)., [Bacchiega T.S](https://www.ncbi.nlm.nih.gov/pubmed/?term=Bacchiega%20TS%5BAuthor%5D&cauthor=true&cauthor_uid=26812352)., et al. *Dogs and Opossums Positive for Vaccinia Virus during Outbreak Affecting Cattle and Humans São Paulo State, Brazil.* Emerg. Infect. Dis.

50. Dutra L.A., de Freitas Almeida G.M., Oliveira G.P., Abrahão J.S., Kroon E.G., Trindade G.S. *Molecular evidence of Orthopoxvirus DNA in capybara Molecular evidence of Orthopoxvirus DNA in capybara (Hydrochoerus hydrochaeris) stool samples.* Arch. Virol. 2017;162:439-48.

51. [Miranda J.B](https://www.ncbi.nlm.nih.gov/pubmed/?term=Miranda%20JB%5BAuthor%5D&cauthor=true&cauthor_uid=28518030)., [Borges I.A](https://www.ncbi.nlm.nih.gov/pubmed/?term=Borges%20IA%5BAuthor%5D&cauthor=true&cauthor_uid=28518030)., [Campos S.P.S](https://www.ncbi.nlm.nih.gov/pubmed/?term=Campos%20SPS%5BAuthor%5D&cauthor=true&cauthor_uid=28518030)., [Vieira F.N](https://www.ncbi.nlm.nih.gov/pubmed/?term=Vieira%20FN%5BAuthor%5D&cauthor=true&cauthor_uid=28518030)., [de Ázara T.M.F](https://www.ncbi.nlm.nih.gov/pubmed/?term=de%20%C3%81zara%20TMF%5BAuthor%5D&cauthor=true&cauthor_uid=28518030)., [Marques F.A](https://www.ncbi.nlm.nih.gov/pubmed/?term=Marques%20FA%5BAuthor%5D&cauthor=true&cauthor_uid=28518030)., et al. *Serologic and Molecular Evidence of Vaccinia Virus Circulation among Small Mammals Serologic and Molecular Evidence of Vaccinia Virus Circulation among Small Mammals from Different Biomes, Brazil.* Emerg. Infect. Dis. 2017;23:931-8.

52. Abrahão J.S., de Souza Trindade G., Pereira-Oliveira G., de Oliveira Figueiredo P., Costa G., Moreira Franco-Luiz A.P., et al. *Detection of Vaccinia virus during an outbreak of exanthemous oral lesions in Brazilian equids.* Equine Vet. J. 2017;49:221-4.

**Supplementary material 2**

**DNA extraction**

For each 100 mg of cheese, we added 900 μl of phosphate-buffered saline (PBS)[49] and 4 units of solid-glass beads (Sigma-Aldrich) in Cryogenics Tubes in Polypropylene. Cheese samples were macerated by using mini-bead beater (Biospec products) and submitted to DNA extraction by using the commercial kit High Pure Viral Nucleic Acid Kit from Roche [76, 124]. To avoid any possibility of laboratory contamination with exogenous DNA, we used purified water and fetal bovine serum as negative controls during DNA extraction process, assuring the quality of analysis.

**Nested PCR and Real-time PCR (qPCR)**

The DNA extracted from all cheese samples were submitted to a nested PCR targeting C11R gene, present in viruses belonging to the genus OPV [80]. For negative and positive controls, we used cheese samples made with milk from experimentally infected cows as previously described by de Oliveira et al. [114]. The amplified fragments were fractionated by 8% polyacrylamide gel electrophoresis (PAGE) at a voltage of 100 V and stainedwith silver. Additionally, were realized reactions to amplify the A56R gene, through the SYBR Green PCR Master Mix (Applied Biosystems, USA) as previously described [51]. The same negative and positive controls were used.

**Phylogenetic analysis**

The amplified C11R fragments were directly sequenced in both orientations and in triplicate by dideoxy method in an ABI3130 platform (Applied Biosystems), and sequence quality was analyzed by using Sequence Scanner Software 1.0 (Applied Biosystems). Sequences were aligned with other reference sequences from the BLAST nucleotide database (http://blast.ncbi.nlm.nih.gov/Blast.cgi) by using MEGA 7.0 (Mega-BACE sequencer, GE Healthcare, Buckinghamshire, UK).

**Support References:**

49. Rehfeld, I.S., et al., *Clinical, hematological and biochemical parameters of dairy cows experimentally infected with Vaccinia virus.* Res Vet Sci, 2013. 95(2): p. 752-7.

51. de Souza Trindade, G., et al., *Real-time PCR assay to identify variants of Vaccinia virus: implications for the diagnosis of bovine vaccinia in Brazil.* J Virol Methods, 2008. 152(1-2): p. 63-71.

76. Abrahão, J.S., et al., *Bovine vaccinia outbreaks: detection and isolation of vaccinia virus in milk samples.* Foodborne Pathog Dis, 2009. 6(9): p. 1141-6.

80. Abrahão, J.S., et al., *Rapid detection of Orthopoxvirus by semi-nested PCR directly from clinical specimens: a useful alternative for routine laboratories.* J Med Virol, 2010. 82(4): p. 692-9.

114. de Oliveira, T.M., et al., *Vaccinia virus is not inactivated after thermal treatment and cheese production using experimentally contaminated milk.* Foodborne Pathog Dis, 2010. 7(12): p. 1491-6.

124. de Oliveira, T.M.L.; Guedes, M.I.M.C.; *Vaccinia virus detection in dairy products made with milk from experimentally infected cows.* Transbound Emerg Dis. 2017 Jun 26. [Epub ahead of print].
